# C3G, through its GEF activity, induces megakaryocytic differentiation and proplatelet formation

**DOI:** 10.1186/s12964-018-0311-5

**Published:** 2018-12-19

**Authors:** Sara Ortiz-Rivero, Cristina Baquero, Luis Hernández-Cano, Juan José Roldán-Etcheverry, Sara Gutiérrez-Herrero, Cristina Fernández-Infante, Víctor Martín-Granado, Eduardo Anguita, José María de Pereda, Almudena Porras, Carmen Guerrero

**Affiliations:** 1 Instituto de Biología Molecular y Celular del Cáncer (IMBCC), Universidad de Salamanca-CSIC, Salamanca, Spain; 2 Instituto de Investigación Biomédica de Salamanca (IBSAL), Salamanca, Spain; 3 Departamento de Bioquímica y Biología Molecular, Facultad de Farmacia, Universidad Complutense de Madrid, Instituto de Investigación Sanitaria del Hospital Clínico San Carlos (IdISSC), Madrid, Spain; 4 Servicio de Hematología y Hemoterapia, Hospital Clínico San Carlos, IdISSC, Departamento de Medicina, Universidad Complutense de Madrid, Madrid, Spain; 5 Departamento de Medicina, Universidad de Salamanca, Salamanca, Spain; 6 Centro de Investigación del Cáncer, Campus Unamuno s/n, Salamanca, Spain

**Keywords:** C3G, Megakaryopoiesis, Megakaryocyte, Platelet, Differentiation

## Abstract

**Background:**

Megakaryopoiesis allows platelet formation, which is necessary for coagulation, also playing an important role in different pathologies. However, this process remains to be fully characterized. C3G, an activator of Rap1 GTPases, is involved in platelet activation and regulates several differentiation processes.

**Methods:**

We evaluated C3G function in megakaryopoiesis using transgenic mouse models where C3G and C3GΔCat (mutant lacking the GEF domain) transgenes are expressed exclusively in megakaryocytes and platelets. In addition, we used different clones of K562, HEL and DAMI cell lines with overexpression or silencing of C3G or GATA-1.

**Results:**

We found that C3G participates in the differentiation of immature hematopoietic cells to megakaryocytes. Accordingly, bone marrow cells from transgenic C3G, but not those from transgenic C3GΔCat mice, showed increased expression of the differentiation markers CD41 and CD61, upon thrombopoietin treatment. Furthermore, C3G overexpression increased the number of CD41^+^ megakaryocytes with high DNA content. These results are supported by data obtained in the different models of megakaryocytic cell lines. In addition, it was uncovered GATA-1 as a positive regulator of C3G expression. Moreover, C3G transgenic megakaryocytes from fresh bone marrow explants showed increased migration from the osteoblastic to the vascular niche and an enhanced ability to form proplatelets. Although the transgenic expression of C3G in platelets did not alter basal platelet counts, it did increase slightly those induced by TPO injection in vivo. Moreover, platelet C3G induced adipogenesis in the bone marrow under pathological conditions.

**Conclusions:**

All these data indicate that C3G plays a significant role in different steps of megakaryopoiesis, acting through a mechanism dependent on its GEF activity.

**Electronic supplementary material:**

The online version of this article (10.1186/s12964-018-0311-5) contains supplementary material, which is available to authorized users.

## Background

C3G, also known as RAPGEF1, is a guanine nucleotide exchange factor (GEF) for several members of the Ras superfamily of GTPases, mainly Rap1 and R-Ras [1, 2]. C3G is essential for mouse embryonic development, since C3G −/− homozygous mice die before embryonic day 7.5 [3]. Embryonic fibroblasts from these mice show impaired cell adhesion, delayed cell spreading and enhanced migration. These defects were rescued by the expression of active Rap1 or R-Ras, suggesting the importance of the GEF function of C3G in cell adhesion, spreading and embryogenesis.

Several studies suggested a role for C3G in cell differentiation. Overexpression of C3G induces neurite outgrowth in rat pheochromocytoma (PC12) and human neuroblastoma (IMR-32) cells [4, 5], and is also required in later stages of neuronal differentiation [6]. This effect of C3G, which is dependent of its catalytic domain, is accompanied by the repression of the cell cycle progression through the induction of the cell cycle inhibitor p21^WAF^ [4]. C3G protein levels increase during the differentiation of monocytes to macrophages [7] and the interaction between Crk and C3G is required for adipocyte differentiation [8]. In addition, C3G overexpression induces myogenic differentiation of mouse mesenchymal C2C12 cells and MEFs, through Akt activation and cyclin D1 repression [9]. Finally, mice expressing a hypomorphic allele for C3G show differentiation deficiencies in a variety of cell types [10].

Megakaryopoiesis is the process by which mature megakaryocytes (MKs) develop from hematopoietic stem cells (HSC). This is a complex process that involves the commitment of HSC to the megakaryocyte lineage, proliferation of progenitors, megakaryocyte (MK) maturation and terminal differentiation to produce platelets (thrombopoiesis) [11]. Immature MKs or megakaryoblasts, undergo changes in their cell surface markers, followed by several cycles of endomitosis to increase their size and DNA content. In addition, immature MKs increase their reservoir of granules and cytoskeletal proteins and form the Invaginated Membrane System (IMS), also known as Demarcation Membrane System o DMS [12]. Mature megakaryocytes then begin the process of shedding their cytoplasm to produce platelets, a complex process that requires the formation of elongated structures called proplatelets.

The Rap1 isoform, Rap1b, is highly expressed in both mature megakaryocytes and platelets, and it is expressed at lower levels in immature megakaryocytes [13]. Rap1b plays an essential role in most platelet functions, including aggregation, coagulation, adhesion and spreading, through activation of the platelet integrin αIIbβ3 [14, 15]. Thrombopoietin (TPO) induces the differentiation of megakaryocytes through its receptor Mpl (myeloproliferative virus ligand). This requires the sustained activation of the Rap1/B-Raf/ERK1/2 pathway [13], the participation of Akt, Jak2 and JNK pathways and the production of ROS [16, 17]. Rap1-mediated activation of the ERK1/2 signaling pathway has been shown to occur via C3G [18]. In fact, although the specific Rap1-GEFs downstream of TPO have not been identified, an association of a CrkL/C3G complex with Mpl has been described [19, 20].

In this line, using transgenic mouse models, our group has established a new role for C3G in platelet activation and aggregation, both in vitro and in vivo [21]. C3G mediates platelet functions triggered by thrombin, ADP, PMA and collagen through the activation of Rap1. In particular, C3G is a mediator of Rap1 activation induced by thrombin and PMA via PKC pathways [21]. The C3G function in platelets is supported by other investigators, who have proposed the formation of a ternary complex, C3G/CrkL/VASP that regulates Rap1b [22]. In addition, we have recently described a role of C3G in platelet secretion, mainly through GEF-independent mechanisms. Thus, transgenic expression of C3G in platelets modulates α-granule exocytosis through interaction with VAMP-7, which results in an overall proangiogenic secretome, facilitating inflammatory angiogenesis and tumor metastasis [23].

Transcription factors of the GATA family play an important role in the regulation of genes involved in MK differentiation. Particularly, GATA-1-binding motifs [(A/T)GATA(A/G)] have been identified in the promoters and/or enhancers of megakaryocyte-specific genes [24].

In this paper we have explored the putative role of C3G in megakaryocyte differentiation, based on (i) its participation in the differentiation of several cell types and (ii) its function in platelet physiology. Our findings indicate that indeed, C3G, whose expression is regulated by GATA-1, is involved in different stages of the megakaryocyte differentiation, from the acquisition of surface markers and ploidy features to the formation of proplatelets.

## Methods

### Transgenic mouse models

Transgenic mice used in this work have been previously described [21]. C3G (full-length) and C3GΔCat (deleted in the catalytic region) transgenes from human origin are expressed under the control of the megakaryocyte and platelet specific PF4 gene promoter. Transgenic C3G (Tg-C3G) lines 2C1 and 6A6 were used. For transgenic C3GΔCat (Tg-C3GΔCat), line 8A3 was used. All mice used in these studies were 8–12 week-old.

### Cell lines and Bone Marrow cell cultures

K562 (ATCC, CCL243) and HEL (ATCC TIB-180) leukemic cell lines were maintained in RPMI-1640 medium supplemented with 10% Fetal Bovine Serum (FBS), 2 mM Glutamine, 100 U/ml Penicillin and 100 μg/ml Streptomycin (Gibco). HEK-293 T (ATCC, CRL-3216, human embryonic kidney) and B16-F10 (ATCC, CRL-6475, murine melanoma) cell lines were grown in DMEM medium (Sigma) containing 10% FBS, 2 mM Glutamine, 100 U/ml Penicillin and 100 μg/ml Streptomycin. The megakaryocytic cell line, Dami, was grown in IMDM medium (Gibco) supplemented with 10% horse serum (HyClone), 20 mM Hepes, 100 U/ml Penicillin and 100 μg/ml Streptomycin (Gibco). Dami cells overexpressing GATA-1 fusioned to V5 and those expressing the empty lentiviral vector pLenti6 V5/LacZ (Gateway) were also grown in this medium but in the presence of 2 μg/ml blasticidine.

Bone marrow cells (BMCs) were obtained from femora and tibiae of transgenic mice by flushing with PBS. The megakaryocytes were enriched by culturing BMCs in IMDM with 10% FBS, streptomycin/penicillin and 50 ng/ml TPO for 5 days. For some experiments, the erythrocytes were removed from the isolated BMCs by incubation, for 2 min on ice, with 2 ml of Red Blood Cell (RBC) lysis buffer (155 mM NH_4_Cl, 10 mM KHCO_3_, 0.1 mM EDTA pH 7.4).

### Megakaryocytic differentiation and maturation in cell lines and BMCs

For MKs differentiation and maturation, cell line cultures were treated with 20 nM phorbol 12-myristate 13-acetate (PMA, Sigma) for 10 days. BMCs were incubated with 50 ng/ml recombinant mouse thrombopoietin (TPO, Miltenyi) in combination with 10 ng/ml Stem Cell Factor (SCF, Miltenyi), 10 ng/ml Interleukin-3 (IL-3, Invitrogen), 10 ng/ml IL-11 (Miltenyi) and 10 ng/ml IL-6 (Miltenyi) for 6 days.

### Cell transfections

K562 cells were permanently transfected with pLTR2-C3G [25], pSuper-C3Gi [26] and CRISPR-C3G (Santa Cruz Biotechnology, Inc.) and the corresponding empty vectors (pLTR2-CT, pSuper-CT and CRISPR-CT), for up-regulation, down-regulation or abrogation of C3G expression, respectively, using the Gene Pulser Electroporation System (Bio-Rad). Additionally, the pLTR2 clones were co-transfected with pSuper.gfp/neo vector to express GFP fluorescence (pLTR2-CT/GFP and pLTR2-C3G/GFP clones). These clones were maintained with complete medium supplemented with 1:20 Killer Hat solution [25], 250 μg/ml neomycin or 2 μg/ml puromycin, as appropriate.

### Lentiviral transduction

The expression of C3G in HEL cells was downregulated by infection with second generation lentiviral particles, which were generated by transient transfection of HEK-293 T cells with psPAX2, pMD2.G and pLVTHM (-CT or -C3Gi) vectors (Addgene), using PEI transfection protocol. HEL cells were infected with the lentiviral particles containing the shRNA of C3G at a MOI of 25. Finally, GFP+ cells were selected by single-cell isolation and clonal expansion. The shRNA used to downregulate C3G expression was: 5′- CCACTATGATCCCGACTAT-3′.

Permanent GATA-1 silencing in Dami cells was performed by infection with human GATA-1 shRNA lentiviral particles (75,000 infectious units) containing a mixture of different shRNAs (Santa Cruz Biotechnology sc-35,452-V) in the presence of 10 μg/ml Polybrene (Santa Cruz Biotechnology sc-134,220). Cells were selected with puromycin (1 μg/ml).

### Cell lysis and Western blot

Cell line cultures were lysed using Cell Lysis buffer (Cell Signaling Technology) supplemented with 25 μM NaF and 1 mM PMSF. Total protein lysates were boiled with 2x Laemmli buffer before being resolved on SDS-PAGE. PVDF membranes were blotted using the following antibodies: C3G H-300 (sc-15,359), p-C3G Tyr504 (sc-12,926), α-globin H-80 (sc-21,005), ERK K-23 (sc-94) and GATA-1 N6 (sc-265) from Santa Cruz Biotechnologies, β-tubulin (T5293) and β-actin (A5441) from Sigma and p21 Waf1/Cip1 (2947) from Cell Signaling Technology.

### Semi-quantitative PCR

RNA was reverse transcribed using SuperScript™ III First-Strand Synthesis System (Thermofisher) to generate DNA. Semi-quantitative PCR was performed using BioTaq™ polymerase (Bioline) using the following primers: for GPA: forward 5´-GGAATTCCAGCTCATGATCTCAGGATG-3′ and reverse 5´-TCCACATTTGGTTTGGTGAACAGATTC-3′; for CD61: forward 5´-TATAGCATTGGACGGAAGGC-3′ and reverse 5´-GACCTCATTGTTGAGGCAGG-3′; for GAPDH: forward 5´-TGCACCACCAACTGCTTAGC-3′ and reverse 5′- TCTTCTGGGTGGCAGTGATG-3′.

### Analysis of cell surface markers and DNA content by flow cytometry

Megakaryocytic markers, CD41 and CD61, and erythroid marker (GPA) were analyzed by flow cytometry using specific fluorochrome-conjugated antibodies: anti-human antibodies for human cell lines from Immunostep (CD41-PE, CD61-APC and GPA-FITC or GPA-PE) and anti-mouse antibodies for mouse BMCs from eBiosciences (CD41-APC and CD61-PE). After differentiation treatment, washed cells were incubated in 100 μl of PBS with 0.5–1 μg of indicated antibodies for 20 min on ice. Then, cells were washed once and the fluorescence was measured using FACSCalibur™ and BD Accuri™ cytometers and analyzed using FlowJo and Accuri software, respectively.

To determine the ploidy status, after 6–10 days of differentiation, cultures of cell lines and BMCs were harvested, washed with PBS and fixed with ethanol by the addition of 0.5 ml of 70% ethanol dropwise. After incubation for at least 1 h, cells were washed twice with PBS and stained with anti-CD41-FITC antibody from BD Pharmigen (BMCs only), as described before, and subsequently incubated with a mixture of 100 μg/ml RNase (Promega) and 50 μg/ml Propidium Iodide (PI, Sigma) in PBS for at least 40 min at RT in the dark. For the analysis, CD41+ cells were gated and cell doublets were excluded from the analysis. The ploidy distribution of the CD41+ populations was determined using a BD Accuri™ cytometer.

### Confocal immunofluorescence microscopy and may-Grunwald-Giemsa staining

3 × 10^6^ cells were seeded onto 6 cm plates containing glass coverslips pre-coated with 5 μg/ml of fibronectin (Sigma). Cells were incubated with serum-free media for 48 h, which allowed for enhanced cell attachment, and then fixed with 3.7% paraformaldehyde (PFA, Sigma) for 15 min. Fixed cells were washed twice with PBS, permeabilized with 0.1% Triton X-100 (Sigma), and washed again with PBS. Coverslips were blocked with 2% BSA for 1 h, and then incubated for 1 h at RT with primary antibody C3G #1008 [25], followed by incubation with Cy5-anti-rabbit antibody. Nuclei were stained with DAPI for 10 min. Coverslips were mounted with Mowiol® (Calbiochem). Images were obtained at the same exposure time with a Leica TCS SP5 confocal microscope and pictures were processed using LSM Image Browser, ImageJ Software and ZEN lite Imaging Software.

For Giemsa staining, after 48 h of PMA treatment, cells were centrifuged onto glass coverslips for 3 min at 500 *g* to allow cell attachment. Then, cells were fixed with 100% ethanol and the slides were stained with May-Grunwald-Giemsa.

### Rap1 activity assay by immunofluorescence

Preparation of samples for detection of active Rap1 by confocal microscopy was performed essentially as described above, with some modifications [27, 28]. Aliquots of 1.5 × 10^6^ cells in PBS were treated with 20 nM PMA at indicated times and then immediately fixed by adding 4% PFA for 20 min at RT. Cells were washed with PBS by centrifugation and permeabilized with 0.2% Triton + 1% BSA for 5 min at RT. Cells were then incubated with 0.3 mg/ml GST-RalGDS-RBD purified protein for 45 min at RT, washed three times with PBS and incubated with anti-GST for 1 h at RT. Cells were washed three times with PBS, and incubated with Cy3 or Cy5-conjugated anti-mouse antibodies for 1 h at RT. Nuclei were stained with DAPI for 10 min. After washing, cells were centrifuged onto glass coverslips for 3 min at 500 *g* and mounted with Mowiol. Negative controls were performed as follows: (1) without the GST-RalGDS-RBD protein, as control of the specificity of the anti-GST primary antibody; (2) by replacing GST-RalGDS-RBD with GST alone at the same molarity; (3) without anti-GST primary antibody, to detect any non-specific staining by the secondary antibody. For active Rap1, z-sections of 0.25 μm were acquired using Leica TCS SP5 confocal microscope. All images were obtained at the same exposure time and processed using LSM Image Browser and ImageJ software.

### CFU-MKs assay

A commercial collagen-based system (MegaCult-C, StemCell Technologies Inc.) was used to assay colony-forming units (CFUs) of mouse megakaryocyte progenitors. Briefly, 2.2 × 10^6^ freshly isolated BM cells were resuspended into 1 ml IMDM medium (33x of the final cell concentration). Then, 50 μl of this cell suspension was mixed with 150 μl of IMDM, containing cytokines at 11x of the final concentration (1x: 50 ng/ml TPO, 20 ng/ml IL-6, 50 ng/ml IL-11 and 10 ng/ml IL-3) and 850 μl of MegaCult™ medium. Finally, 600 μl of cold collagen was added (≈1600 μl) and 750 μl of this final cell suspension was cultured into the two wells of a double chamber slide (μ-Slide 2 well, Ibidi), each containing ≈50,000 cells. Cultures were maintained at 37 °C and 5% CO_2_ for 8 days. Collagen gels were dehydrated, fixed and stained according to the manufacturer’s specifications. Acetylchorinesterase-positive colonies with 3 or more MKs were scored as CFU-MKs.

### Time lapse analysis of bone marrow explants

Intact marrow was obtained by flushing mouse femora with Tyrode’s-HEPES buffer (134 mM NaCl, 0.34 mM NaHPO_4_, 2.9 mM KCl, 12 mM NaHCO_3_, 20 mM HEPES), 5 mM Glucose, 0.35% Albumin, 1 mM MgCl_2_, 2 mM CaCl_2_ and 10 U/ml Penicillin/Streptomycin) using a 21-gauge needle. The marrows were cut into 0.5–1 mm thick transverse sections with a surgical blade, under a binocular microscope. The explants were placed in an incubation chamber (μ-Slide 8 well IbiTreat, Ibidi) with Tyrode’s-HEPES buffer containing 5% mouse serum and were maintained at 37 °C for 6 h. MKs at the periphery of the explant were monitored under an inverted microscope (Nikon Eclipse TE2000-E), coupled to a video camera (Hamamatsu Orca-er). The images were sequentially acquired at 10 min intervals for 6 h and then mounted and processed using ImageJ and Metamorph software.

To identify the MKs in the periphery of the explant, anti-mouse CD41-APC antibody (eBiosciences) was added to the Tyrode’s-HEPES buffer prior to placing the explants in the incubation chamber. MKs were classified according to the morphology: i) spherical megakaryocytes, ii) megakaryocytes with protrusion and iii) megakaryocytes with proplatelets [29].

### Isolation of cells from the bone matrix

To analyze the MKs associated to the osteoblastic niche, after isolation of BMCs from the femur, the remaining bones were cut into 1 mm pieces and incubated with 1 mg/ml collagenase Type I (Sigma) and 1 mg/ml dispase Type II (Sigma) at 37 °C for 2 h under vigorous stirring to detach the cells most tightly adhered to the bone matrix.

### Analysis of the number of platelets by flow cytometry

Blood (100–200 μl) was collected by submandibular puncture from anesthetized mice (isoflurane), and anticoagulated with EDTA. Blood was washed with same volume of Tyrode’s-HEPES buffer plus 5 mM glucose, and stained with anti-CD41-APC antibody for 15 min at RT. The number of platelets was determined by measuring 50 μl of blood using a BD Accuri ™ cytometer.

### Platelet production in vivo in response to TPO

Mouse TPO (Molecular Innovations®) was administered by intraperitoneal injection (5 μg per mouse). Platelet number was determined, at the indicated time points, as described above. Bone marrow cells were harvested 11 days after injection and the percentage of megakaryocytes was analyzed by flow cytometry.

### Platelet clearance analysis

Mice were injected, through the lateral tail vein, with 600 μg of hydroxysuccinimido-biotin (NHS-biotin, Sigma) in 200 μl of buffer containing 140 mM NaCl and 10% DMSO. At the indicated time points, 20 μl of whole blood, from submandibular puncture, was mixed with 200 μl ml BSGC buffer (116 mM NaCl, 13.6 mM tri-sodium citrate, 8.6 mM Na_2_HPO_4_, 1.6 mM KH_2_PO_4_, 0.9 mM EDTA, 11,1 mM glucose) and 1 ml of balanced salt solution (BSS; 149 mM NaCl, 3.7 mM KCl, 2.5 mM CaCl_2_, 1.2 mM MgSO_4_, 7.4 mM HEPES, 1.2 mM KH_2_PO_4_, 0.8 mM K_2_HPO_4_, 3% FBS). Cells were pelleted at 1400 *g* for 5 min and resuspended in PBS. Platelets were stained with CD41-APC for 15 min, followed by PE-Streptavidin for 1 h on ice. Biotinylated platelets were washed in BSS buffer and analyzed by flow cytometry.

### Tumor implantation and analysis of platelets, MKs and adipocytes

1 × 10^6^ B16-F10 melanoma cells in PBS were subcutaneously injected into Tg-C3G and WT-C3G mice. After 15 days of tumor growth, the BM was extracted, fixed in 4% formaldehyde, embedded in paraffin and stained with H&E. The number of MKs was quantified using an Ariol IHC Scanner, based on the Area_score and the Intensity_score parameters (Leyca Biosystems). The number of adipocytes was determined using the Adiposoft software (ImageJ), which discriminates particles between 4 and 30 μm. A visual double-check was done to eliminate the false-positive results.

### Statistical analysis

Data have been represented as the mean ± SEM (Standard Error of the Mean) or the median ± SEM values of at least 3 independent experiments from each genotype. The Kolmogorov-Smirnov test was performed to determine if data fit into a normal distribution. To compare between two experimental groups, unpaired Student’s t-test was computed, when the data were normally distributed. The Mann Whitney’s U-test was computed as a non-parametric procedure when our data were not normally distributed.

In fluorescence measurements in cell lines cultures, due to high variability in fluorescence intensities between independent experiments, the data were normalized against the control values. To calculate the significance between the different experimental conditions, a two-way ANOVA test was performed. Then, Holm-Sidak post-hoc pairwise analysis was calculated to determine the significant differences in groups two to two.

## Results

### C3G induces the acquisition of MK features, such as MK-like morphology and expression of MK surface markers in K562 and HEL cell lines

**Fig. 1:**
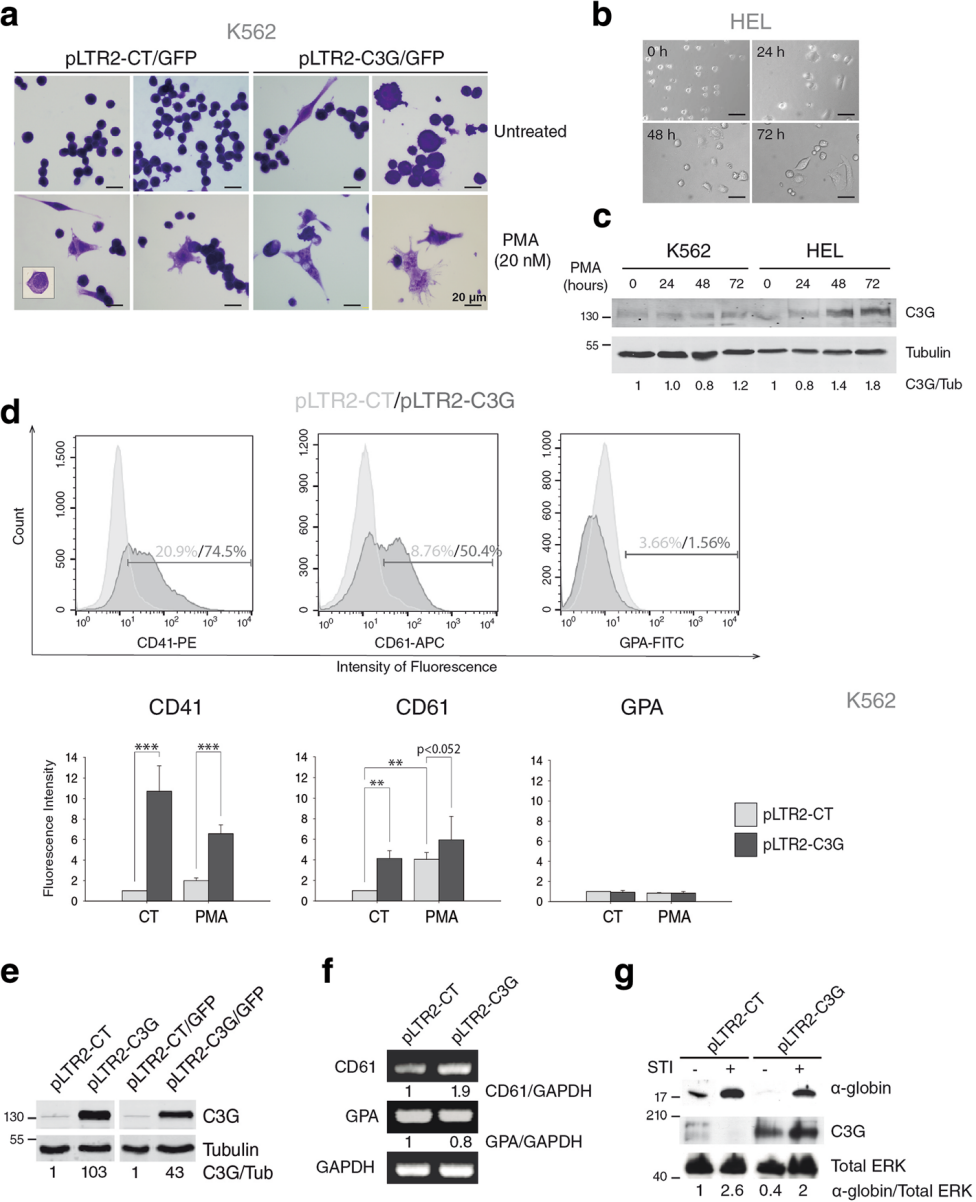
Overexpression of C3G in K562 cells increases megakaryocytic markers. **a** May-Grünwald-Giemsa staining of the indicated K562 clones untreated or treated with 20 nM PMA for 72 h. Scale bars: 20 μm. **b** Representative images showing the morphology of HEL cells after 24, 48 and 72 h of PMA treatment. Images were obtained using a Zeiss Axiovert 135 inverted microscope. Scale bars: 50 μm, **c** Analysis of C3G expression in K562 and HEL cells stimulated with 20 nM PMA at the indicated times. Tubulin was used as loading control. C3G expression values are relative to non-treated cells and normalized against tubulin (C3G/Tub). **d** Expression of CD41, CD61 and GPA markers was analyzed by flow cytometry using specific fluorochrome-conjugated antibodies (CD41-APC, CD61-PE and GPA-FITC). Representative flow cytometry plots of untreated cells are shown. Histograms represent the mean ± SEM of the fluorescence intensity (relative units) of CD41, CD61 and GPA from at least 4 independent experiments of each clone, treated as indicated. 2-way ANOVA and Holm-Sidak analysis were done. **p < 0.01, ***p < 0.001. **e** Representative Western blots showing C3G expression in the pLTR2 and pLTR2/GFP clones. The numbers represent C3G expression values relative to empty vectors and normalized against tubulin (C3G/Tub), which was used as loading control. **f** Analysis of CD61 and GPA expression in untreated K562 clones, pLTR2-CT and pLTR2-C3G, by RT-PCR. The numbers represent expression values relative to pLTR2-CT expressing cells and normalized against GAPDH, which was used as housekeeping gene. **g** Western blot analysis of α-globin and C3G in the indicated clones of K562, treated or not with 2 μM STI-571, an inducer of erythroid differentiation in these cells, [46]. The expression of total ERK was used as loading control. α-globin/ERK ratios are shown. All values are relative to control, non-treated cells. STI: STI-571 (Imatinib mesylate); CT: control

K562 and HEL are two cell lines with erythroid characteristics widely used to study megakaryocytic differentiation, since they can acquire megakaryocytic features upon stimulation with TPO or phorbol esters. The first evidence of the implication of C3G in MK differentiation was the observation of a high proportion of K562 cells, stably transfected with hC3G, with a megakaryocytic-like morphology, i.e. large vacuolated cells with several membrane extensions (Fig. 1a and Additional file 1: Figure S1a). This morphology was similar to the acquired by non-transfected K562 and HEL cells, upon PMA stimulation (Fig. 1a & b). Moreover, the expression of C3G increased during PMA-induced MK differentiation, mainly in HEL cells, which suggests an implication of C3G in this process (Fig. 1c). However, silencing of C3G expression did not modify the morphology of PMA-treated K562 cells (Additional file 1: Figure S1a & 1b).

Based on the above results, we analyzed the expression on the surface of the megakaryocyte markers CD41 and CD61 in K562 clones with overexpression or silenced expression of C3G. C3G overexpression per se induced a significant increase in CD41 and CD61 levels in two different clones, pLTR2-C3G (Fig. 1d & e) and pLTR2-C3G/GFP (Additional file 1: Figure S1c). Increased expression of CD41 and CD61 in the pLTR2-C3G clone was coupled to a slight decrease in the expression of the erythroid marker GPA (Fig. 1d), which was confirmed by RT-PCR (Fig. 1f). Accordingly, the expression of the erythroid marker α-globin was highly decreased upon C3G overexpression (Fig. 1g). Remarkably, the levels of CD41 and CD61 induced by C3G overexpression were similar to those induced by PMA treatment in control cells. PMA did not further increase the levels of these MK markers induced by C3G overexpression (Fig. 1d), indicating that C3G would participate in the PMA-induced differentiation pathway.

On the other hand, C3G silencing or ablation did not change the expression of these surface markers (Additional file 2: Figure S2a & 2b), although an increase in GPA expression, coupled to a slight decrease in CD61 expression was observed by RT-PCR (Additional file 2: Figure S2c). The increase in GPA expression was also detected by flow cytometry (Additional file 2: Figure S2a). Altogether, these results suggest that C3G is involved in the induction of the expression of megakaryocytic differentiation markers in K562 and HEL cells, thus contributing to the commitment of these cells to the megakaryocytic lineage.

### C3G regulates the ploidy status of K562 and HEL cell lines during MK differentiation

**Fig. 2:**
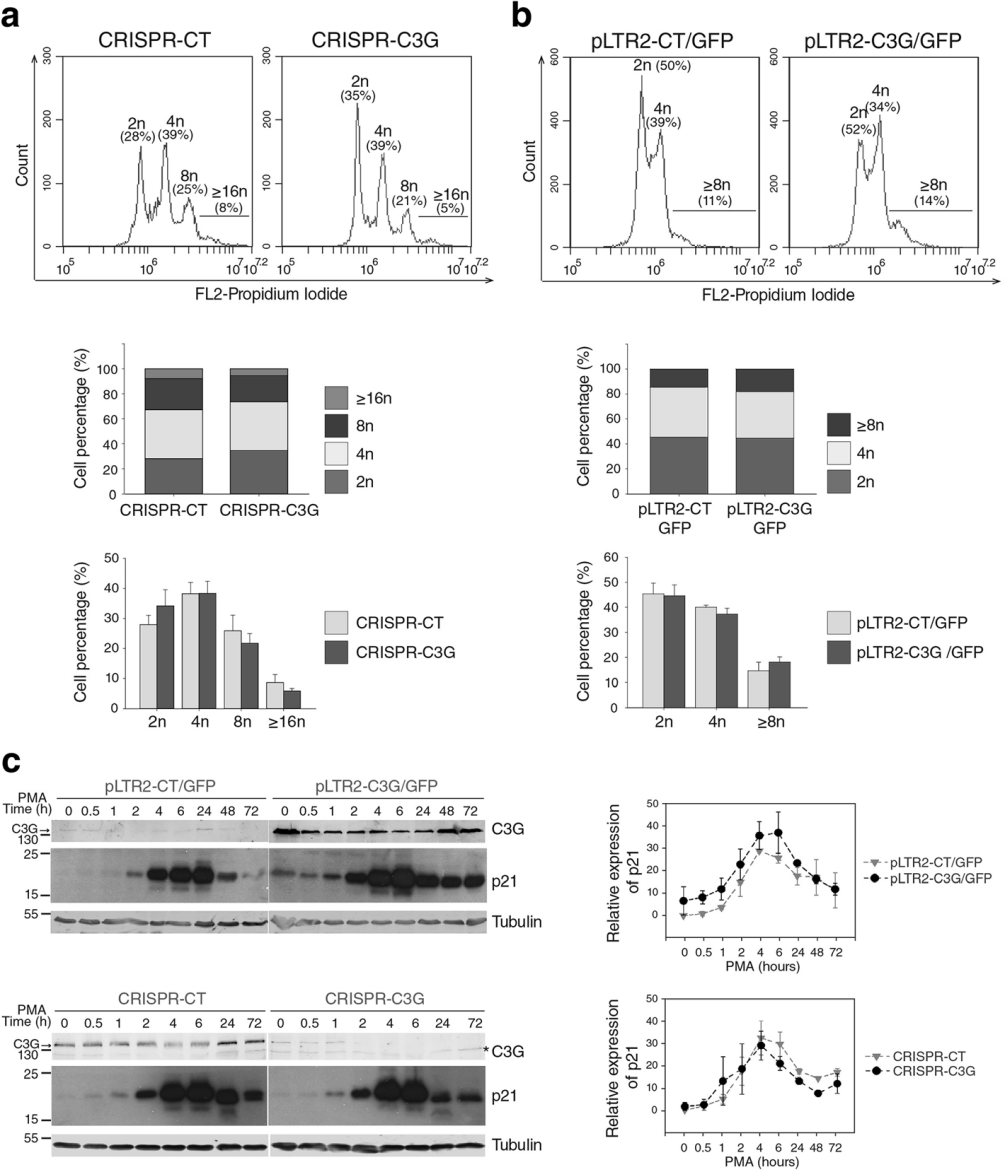
Overexpression of C3G in K562 increases polyploidization and p21 expression. The polyploidization state of K562-pLTR2/GFP and -CRISPR clones was identified by propidium iodide staining after 10 days of differentiation with 20 nM PMA. Representative flow cytometry plots of ploidy distribution, stacked bars and histograms corresponding to CRISPR clones (**a**) and pLTR2/GFP clones (**b**). We identified up to 4 different populations: diploid (2n), tetraploid (4n) and polyploidy cells (8n and ≥ 16n), indicating the percentage of cells corresponding to each population. Stacked bar histograms represent the mean of the percentage of cells of each genotype belonging to the different ploidy populations. Histograms represent the mean ± SEM of the quantification of the percentage of individual ploidy population. **c** Time course Western blot analysis of the expression of C3G (indicated by an arrow) and p21 in K562 cells transfected with pLTR2/GFP plasmids (upper panels) or CRISPR plasmids (lower panels) treated with PMA (20 nM) for the indicated times. Left panel: representative images of Western blot. Tubulin was used as loading control. The asterisk indicates a non-specific band. Right panel: Line/scatter plots of p21 expression. Values are normalized with tubulin and relativized to control, non-treated cells (*n* = 2). The ANOVA analysis indicates that there are statistically significant differences in p21 expression between pLTR2-CT and pLTR2-C3G at the different time points (*p* = 0.019)

At the end of the proliferation phase, megakaryocyte precursors exit the normal cell cycle and undergo endomitosis. In order to establish the role of C3G in this phase of the MK differentiation we determined the ploidy status induced by PMA in our K562 and HEL clones. As shown in Fig. 2a, the percentage of polyploid cells decreased in C3G-KO K562 cells, as compared to control cells. This was accompanied by an increase in the percentage of diploid cells. Reciprocally, the percentage of polyploid cells was higher in the C3G-overexpressing K562 cells, as compared to their controls (Fig. 2b).

The polyploidization of early MK progenitors requires cell cycle arrest and entry into the endomitosis cycle [30, 31]. p21^Waf-1/Cip-1^ (p21) is a potent inhibitor of cyclin-dependent kinases (CDKs) involved in cell cycle arrest of various cell types. p21 has been shown to be highly expressed in mature megakaryocytes, suggesting an important role of this protein in the proliferative arrest of cells during MK differentiation [32].

With all these considerations, we analyzed the expression of p21 in our K562 clones with C3G overexpression (pLTR2-C3G/GFP) or ablation (CRISPR-C3G). Figure 2c shows a clear increase in p21 expression throughout the treatment with PMA, which was maximum between 4 and 24 h of stimulation. Interestingly, C3G-overexpressing K562 cells showed a ten-fold increase in p21 expression under non-stimulation conditions, as compared to control cells. In addition, these pLTR2-C3G/GFP cells maintained higher sustained levels of p21 throughout the time course. Moreover, decreased levels of p21 were observed in CRISPR-C3G cells after 24 h of PMA treatment, although in this case the data were not significant. This result indicates that C3G would modulate the expression of p21, thus contributing to cell cycle arrest.

### PMA-induced C3G phosphorylation promotes a sustained activation of Rap1

**Fig. 3:**
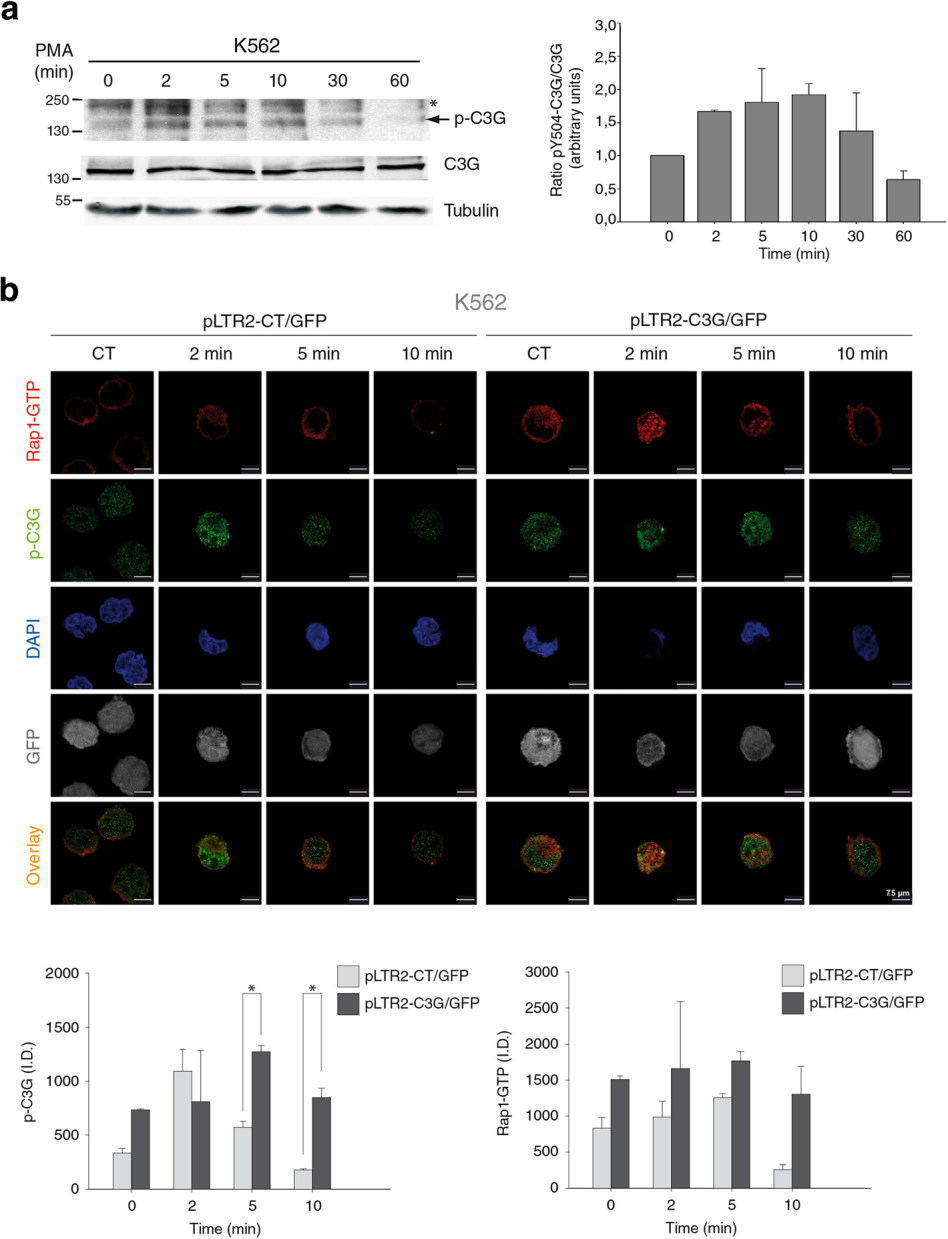
PMA induces C3G phosphorylation in K562 cells, which correlates with a sustained Rap1 activation. **a** Left panel: time course Western blot analysis of phospho-Y504-C3G (p-C3G) expression in non-transfected K562 cells treated with 20 nM PMA for 2, 5, 10, 30 and 60 min. The expression of total C3G and tubulin were used as loading controls. The asterisk indicates a non-specific band. Right panel: Histogram showing pY504-C3G/C3G ratios, relativized to control, non-treated cells. **b** Representative immunofluorescence confocal microscopy images of the indicated K562 clones treated with 20 nM PMA for 2, 5 and 10 min, fixed, permeabilized and incubated with: anti-phospho-C3G/anti-rabbit Cy3 antibodies (green), purified GST-RalGDS-RBD and anti-GST/anti-mouse Cy5 antibodies (red) to detect active Rap1-GTP, and DAPI (blue). The overlay images are made without DAPI channel. Images of GFP expression are shown. Histograms represent the mean ± SEM of the integrated density (I.D.) of p-C3G (left panel) and Rap1-GTP staining (right panel). The ANOVA analysis indicates that there is a significant variability between pLTR2-C3G and pLTR2-CT in the fluorescence intensities of p-C3G and Rap1-GTP, independently of the treatment used (*p* < 0.05). In addition, Holm-Sidak method was done. *p < 0.05

Since activation of C3G by phosphorylation on tyrosine 504 is required to carry out some of its functions [33], we analyzed the phosphorylation status of C3G in K562 cells treated with 20 nM PMA at several time points (2, 5, 10, 30 and 60 min), using a specific anti-phospho-Tyr 504 antibody. Indeed, PMA induced a transient increase in C3G phosphorylation in K562 cells (Fig. 3a). Whereas in control, pLTR2-CT/GFP-expressing, cells phospho-C3G levels peaked at 2 min of treatment with PMA, C3G-overexpressing cells showed increased basal levels that were maintained throughout the time course (Fig. 3b). Moreover, treatment with PMA induced an increase in Rap1-GTP levels in cells with endogenous C3G expression that were maximal between 2 and 5 min of stimulation and decreased after 10 min. This indicates that PMA promotes a transient activation of Rap1, as described by other authors [15]. However, overexpression of C3G induced a more sustained activation of Rap1, in correlation with the sustained phosphorylation of C3G. Negative controls of the immunofluorescence Rap1 activation assay are shown in Additional file 3: S3. Overall, these results indicate that C3G overexpression increases PMA-induced Rap1 activation and that phosphorylation of C3G in Y504 is important for this effect.

### GATA-1 regulates C3G expression during MK differentiation

**Fig. 4:**
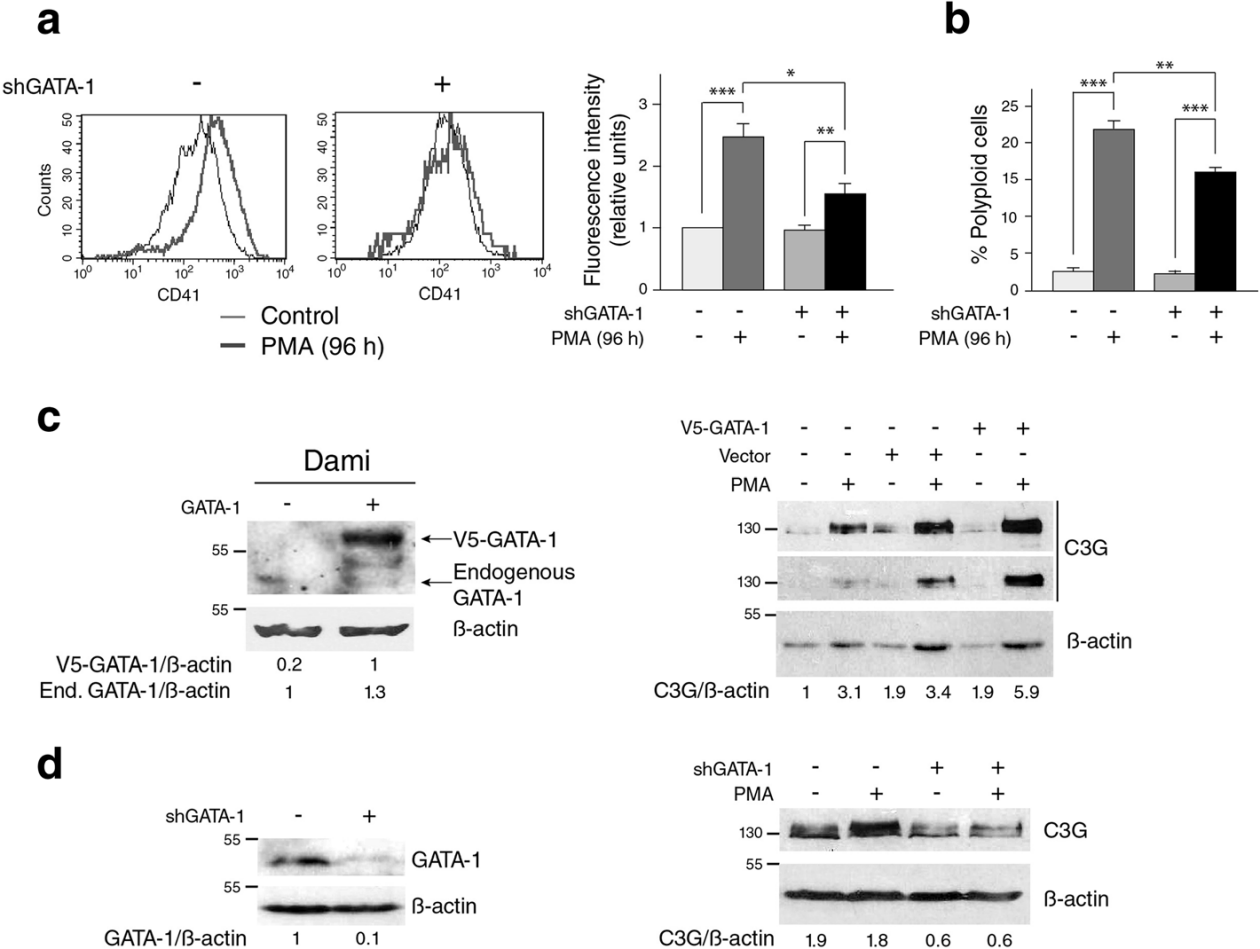
GATA-1 regulates C3G expression in Dami cells. **a** Dami cells with a permanent GATA-1 knockdown (shGATA-1) or overexpression of the fused protein V5-GATA-1 (a gift from Dr. Laura Gutiérrez, University of Oviedo), as well as their control cells (expressing empty vectors) were used. Representative flow cytometry plots of the expression of CD41 marker in Dami cells treated with 100 ng/ml PMA for 96 h. Histograms represent the mean ± SEM of the fluorescence intensity (relative units) of CD41 from 3 to 5 independent experiments of each clone. **b** Histograms represent the mean ± SEM of the percentage of polyploidy (>4n) Dami cells stimulated with PMA for 96 h (*n* = 5). **c** Ectopic expression of GATA-1 in Dami cells induces C3G expression. Left panel: Western blot showing the expression of V5-GATA-1 protein or endogenous GATA-1 in Dami cells. V5-GATA-1/β-actin or endogenous (End.) GATA-1/β-actin ratios are shown. Values were normalized against endogenous GATA-1 levels in cells transfected with the empty vector. Right panel: C3G expression in Dami cells transfected with V5-GATA-1 or its control, stimulated with 100 ng/ml PMA for 96 h. Normalized C3G/β-actin ratios are shown. **d** Silencing of GATA-1 abolished PMA-induced C3G expression in Dami cells. Left panel: Western blot showing GATA-1 levels in Dami cells. Values were normalized using β-actin. Righ panel: C3G expression in Dami cells with silenced GATA-1 expression and treated with 100 ng/ml PMA for 96 h. Normalized C3G/β-actin ratios are shown

The hematopoietic transcription factor GATA-1 induces the expression of genes involved in MK differentiation. Accordingly, the silencing of GATA-1 in Dami cells prevented, both the expression of CD41 and the polyploidization induced by PMA (Fig. 4a & b). A ChIP analysis suggested the existence of binding sequences for GATA-1 at evolutionary conserved sites corresponding to C3G gene regulatory regions (Additional file 4: Figure S4a-c). Therefore, GATA-1 could be a potential regulator of C3G during megakaryocytic/platelet differentiation. Indeed, overexpression of GATA-1 in Dami cells increased C3G expression during PMA-induced MKs maturation (Fig. 4c). Consequently, GATA-1 silencing prevented the increase in C3G expression under PMA treatment (Fig. 4d). Therefore, C3G would be a newly identified mediator of GATA-1 involved in MK differentiation.

### Transgenic C3G expression increases the proportion of poliploid/mature megakaryocytes in primary bone marrow cultures

The production of megakaryocytes from adult HSC of bone marrow (BM), cultured in the presence of TPO and a cocktail of cytokines, is an ex vivo model commonly used to study the mechanisms involved in the expansion and differentiation of megakaryocytic progenitors. To study, in this ex vivo model, the participation of C3G in megakaryopoiesis, we have used two lineages of transgenic mice for C3G (2C1 and 6A6) and one for C3GΔCat (8A3 lineage), where the transgenes are expressed under the PF4 gene promoter [21, 23].

**Fig. 5:**
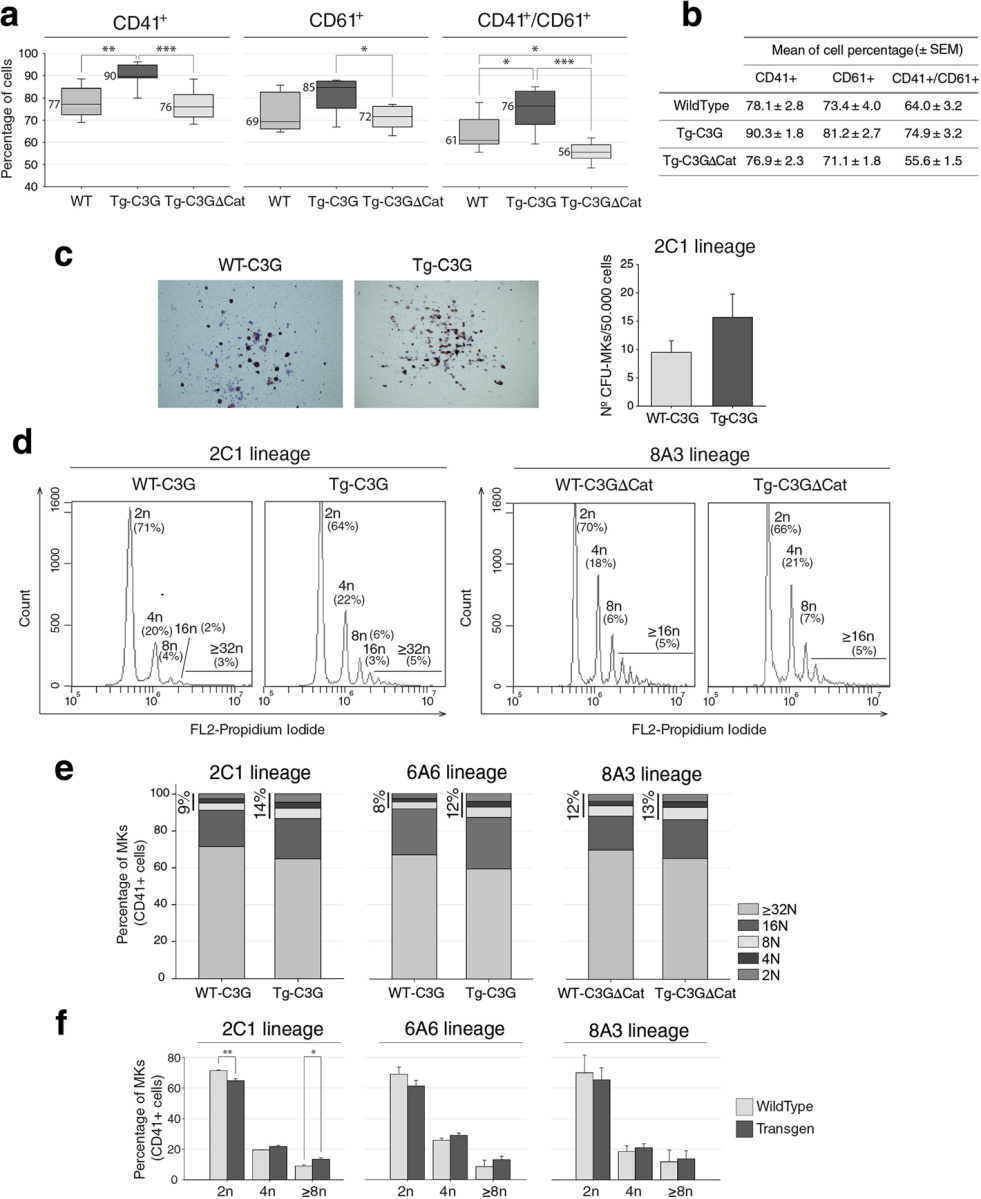
Tg-C3G expression promotes CFU-MKs and increases the percentage of mature megakaryocytes in BM in vitro. **a** Freshly isolated BM cells were cultured with TPO for 6 days. The percentage of CD41+, CD61+ and double CD41+/CD61+ cells was analyzed by flow cytometry. Box plots represent the median ± SEM of the percentage of positive cells of 6 different measures from three independent cultures of each genotype. Mann-Whitney U test was done. *p < 0.05, **p < 0.01 and ***p < 0.001. **b** Table indicating the mean ± SEM of the percentage of positive cells of each genotype. **c** Representative images of a CFU-MK from WT-C3G and Tg-C3G (2C1 lineage) BMs. Histograms represent the mean ± SEM of the total number of CFU-MKs in BM cultures from 3 mouse of each genotype and 2 cultures per mouse. **d** Representative flow cytometry plots of ploidy distribution of FSChigh/CD41+ MKs (WT and Tg). We identified up to 5 different populations; 2n, 4n, 8n, 16n and ≥ 32n. The percentage of FSChigh/CD41+ MKs corresponding to each population is indicated. **e** Stacked bar histograms of the percentage of MKs of each genotype belonging to the different ploidy populations (2n, 4n, 8n, 16n and ≥ 32n) of BM cells. Vertical bars indicate the percentage of cells with a DNA content ≥8n. **f** Histograms represent the mean ± SEM of the percentage of individual ploidy populations of BM cells. Data correspond to 3 different experiments from 3 mouse of each genotype. Data were analyzed using the t-test. *p < 0.05 and **p < 0.01

BM cultures from Tg-C3G mice harbored a higher percentage of megakaryocytes than WT or Tg-C3GΔCat mice, according to the statistically significant increase in the CD41+ and double CD41+/CD61+ populations. Moreover, there was a significant decrease in the percentage of CD41+/CD61+ megakaryocytes in bone marrow cultures derived from Tg-C3GΔCat mice, as compared to WT and Tg-C3G cells (Fig. 5a & b). In agreement with these results, although data did not reach significance, we found a greater number of acetylcholine-positive megakaryocyte colonies in BM cultures from Tg-C3G mice, in comparison with their WT controls (Fig. 5c).

Overall, these results indicate that C3G plays a positive role in the acquisition of megakaryocytic markers upon TPO stimulation and increases the ability of MK progenitors to proliferate and generate MK colonies. This function of C3G is dependent on its catalytic, GEF activity, in agreement with the results found in human cell lines.

Next, we evaluated the ploidy status of CD41+ BM cell population, obtained after 6 days of culture (Fig. 5d-f). A significant increase in CD41+ cells with a DNA content ≥8n was observed in the Tg-C3G 2C1 lineage (14% in Tg-C3G vs 9% in WT), which was coupled to a significant decrease in 2n CD41+ cells (64% in Tg-C3G vs 71% in WT). A similar tendency was observed in the 6A6 lineage (≥8n population: 12% in Tg-C3G vs 8% in WT). These results indicate that overexpression of C3G in megakaryocytes induces a higher rate of DNA replication. To verify these results, polyploidization was analyzed in mature CD41+ megakaryocytes of the Tg-C3GΔCat mice. The results indicate a DNA distribution in mature Tg-C3GΔCat megakaryocytes similar to that of the WT (12% in Tg-C3GΔCat vs 13% in WT), indicating that overexpression of this mutant did not affect endomitosis.

Altogether, these results support a role for C3G in the acquisition of polyploid features, which seems to be dependent on its GEF activity.

### C3G promotes megakaryocyte migration and proplatelet formation

**Fig. 6:**
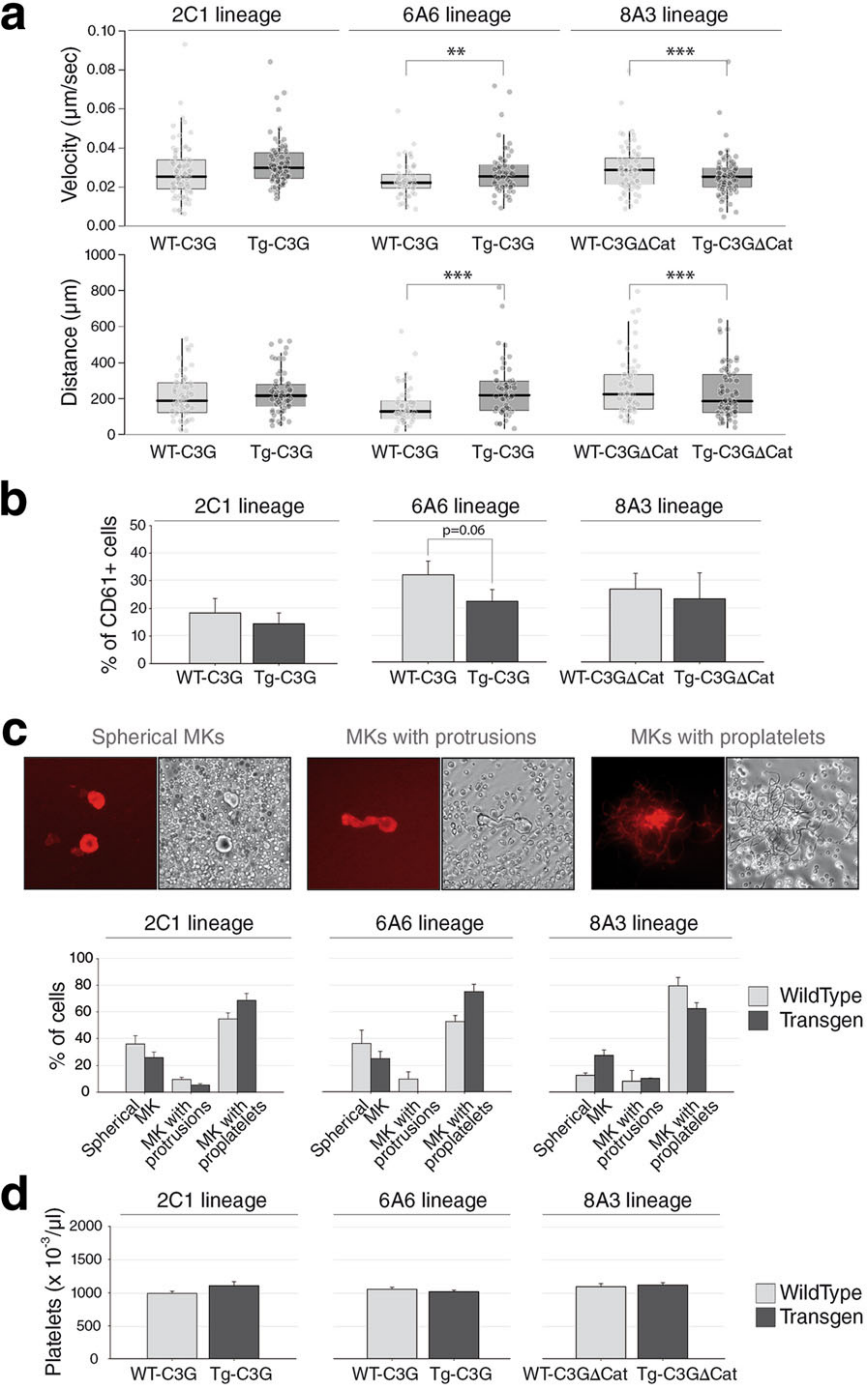
C3G regulates megakaryocyte motility and promotes the formation of proplatelets. **a** Transverse sections of bone marrows from the different genotypes were plated in an incubation chamber and maintained at 37 °C for 6 h. MKs at the periphery of the explants were tracked under the microscope and images were acquired at 10 min intervals. Upper box plots represent the median ± SEM velocity (μm/second) of individual megakaryocytes 6 h after their release from bone marrow explants. Lower box plots represent the median distance (μm) covered by megakaryocytes from bone marrow explants. Three mice of each genotype were analyzed. Mann Whitney U test were done. **p < 0.01, ***p < 0.001. **b** Percentage of MKs most strongly associated to the osteoblastic niche. After extraction of the bone marrow, small pieces of femur were treated with collagenase and dispase for 2 h and the percentage of CD61+ cells was analyzed by flow cytometry. Mann-Whitney U test was done. **c** Representative images of the different stages of MK maturation: spherical megakaryocytes, megakaryocytes with extending protrusions and megakaryocytes with proplatelets. Fluorescence microscope images (Left panels) and brightfield inverted microscope images (right panels). The histograms represent the mean ± SEM of the percentage of cells of each phenotype measured in two different experiments, with at least 4 explants from each genotype. **d** C3G did not modify platelet count. Counts were performed in peripheral blood collected from 6-month mice of the different genotypes using Hemavet Counter HV950FS. The histograms represent the mean ± SEM of the number of platelets. Mann-Whitney U test was done, but no significant differences were observed between Tg-C3G or Tg-C3GΔCat vs WT.

Interaction of megakaryocytic progenitors with the BM microenvironment is essential for proper megakaryopoiesis and subsequent development of megakaryocytes into platelets. Upon physiological platelet demand, megakaryocyte progenitors migrate from the osteoblastic niche toward the vascular niche in a process mediated by the integrin αIIbβ3 [34]. To determine whether C3G regulates megakaryocyte motility, we analyzed the migration capacity of mature MKs in bone marrow explants of the different genotypes in terms of migration velocity and distance covered. As shown in Fig. 6a (upper box plots), the migration velocity of MKs released from the bone marrow of mice of the 6A6 lineage was significantly higher in the Tg-C3G than in the WT explants. Similar results were obtained with the 2C1 lineage, although in this case differences were not statistically significant. On the other hand, Tg-C3GΔCat MKs showed a significant decrease in motility, compared to their WT MKs (8A3 lineage). These data correlate with the results derived from the analysis of the distance covered (Fig. 6a, lower box plots).

In vivo proplatelet formation by megakaryocytes is temporarily regulated by interactions with the extracellular matrix. In particular, interaction with type I collagen, which is particularly abundant in the osteoblastic niche, strongly inhibits proplatelet formation. To analyze the interaction of our transgenic megakaryocytes with the extracellular matrix of the bone marrow, we studied the megakaryocyte progenitors most strongly associated with the bone matrix, i.e. those that are only release from the bone after treatment with collagenase and dispase. Figure 6b shows that C3G overexpression (Tg-C3G 6A6 lineage) modestly decreases the percentage of MK progenitors that are bound to the bone matrix, whereas overexpression of the C3GΔCat mutant showed no effect. These data suggest that C3G could play a role in the mobilization of mature megakaryocytes from the osteoblastic niche to the vascular niche.

Next, we studied whether the increased production and mobilization of MK, induced by C3G, was reflected in a greater formation of proplatelets. BM explants were maintained in a physiological buffer in the presence of anti-CD41-APC antibody, and MK morphology was monitored for 6 h by time-lapse microscopy. Results in Fig. 6c showed a 10 and 20% increased proportion of megakaryocytes extending proplatelets in explants from Tg-C3G mice of the 2C1 and 6A6 lineages respectively, compared to their WT. The implication of the catalytic domain of C3G in this effect was verified by the decrease of about 20% in the ability of the Tg-C3GΔCat MKs to form proplatelets, as compared to its control (8A3 lineage). These results indicate that the overexpressed C3G, through its catalytic domain, promotes the formation of proplatelets in mature megakaryocytes. The Additional file 7: Video S1 was used to determine the speed and the mobility of each megakaryocyte from the explant.

The above results suggest that C3G may induce the formation of a greater number of platelets. However, platelet count from peripheral blood, performed with a Hemavet Counter, revealed no differences between the different genotypes (Fig. 6d). Additionally, no differences were found in the platelet count between males and females (data not shown). The analysis of the platelet half-life excluded the possibility of a differential platelet turnover in the different genotypes (Additional file 5: Figure S5a & b). Moreover, no differences were detected in the mRNA or protein content between transgenic and wild-type platelets (data not shown).

### Platelet C3G increases platelet counts and induces adipogenesis in BM under pathological conditions

**Fig. 7:**
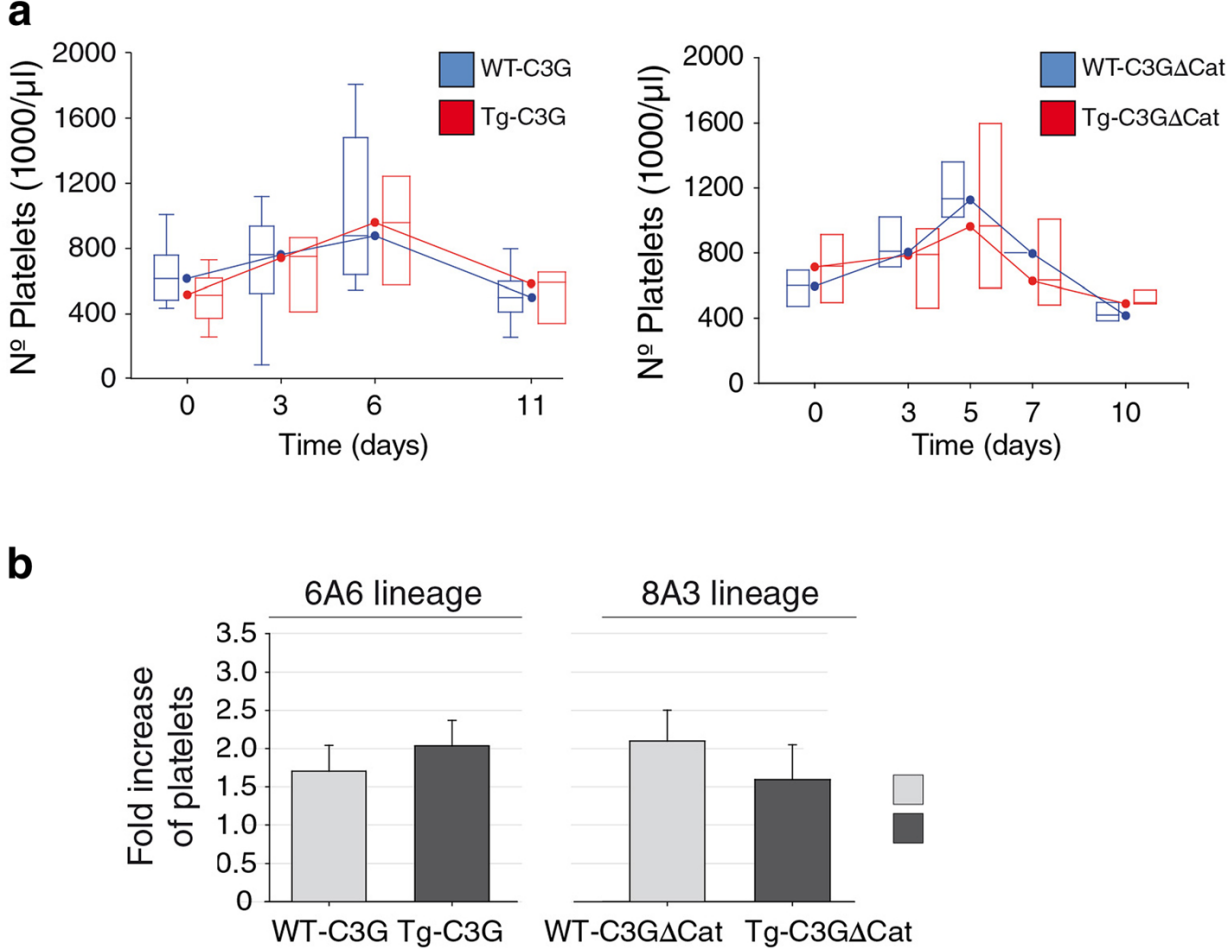
C3G increases platelet production in response to TPO. Mice were injected intravenously with 5 μg TPO/mouse and blood samples were collected at the indicated time points. **a** Time course of platelet counts in Tg-C3G (left panel), Tg-C3GΔCat (right panel) and their corresponding wild-types. The graphs represent the median of the number of platelets (1000/μl) of each phenotype. **b** The histograms represent the mean ± SEM of the maximum fold change in the number of platelets in blood after TPO treatment (platelet count at day 5–6 /platelet count at day 0)

Considering the unexpected similar platelet counts obtained in vivo, we analyzed the production of platelets under pathological conditions. For that, we used two approaches that mimic a pathological thrombopoiesis: TPO injection and tumor implantation. Mice were treated with a single intraperitoneal injection of 5 μg TPO, and blood was collected at different days post-injection to analyze platelet production. The administration of TPO increased the platelet count, which peaked at day 5–6, both in transgenic mice and in their controls (Fig. 7a & b). There was a slight increase in the fold change of platelet number in Tg-C3G compared to WT, which correlated with a slight decrease in Tg-C3GΔCat versus WT. These results could indicate a tendency of C3G to increase the production of platelets in a stress situation in vivo.

**Fig. 8:**
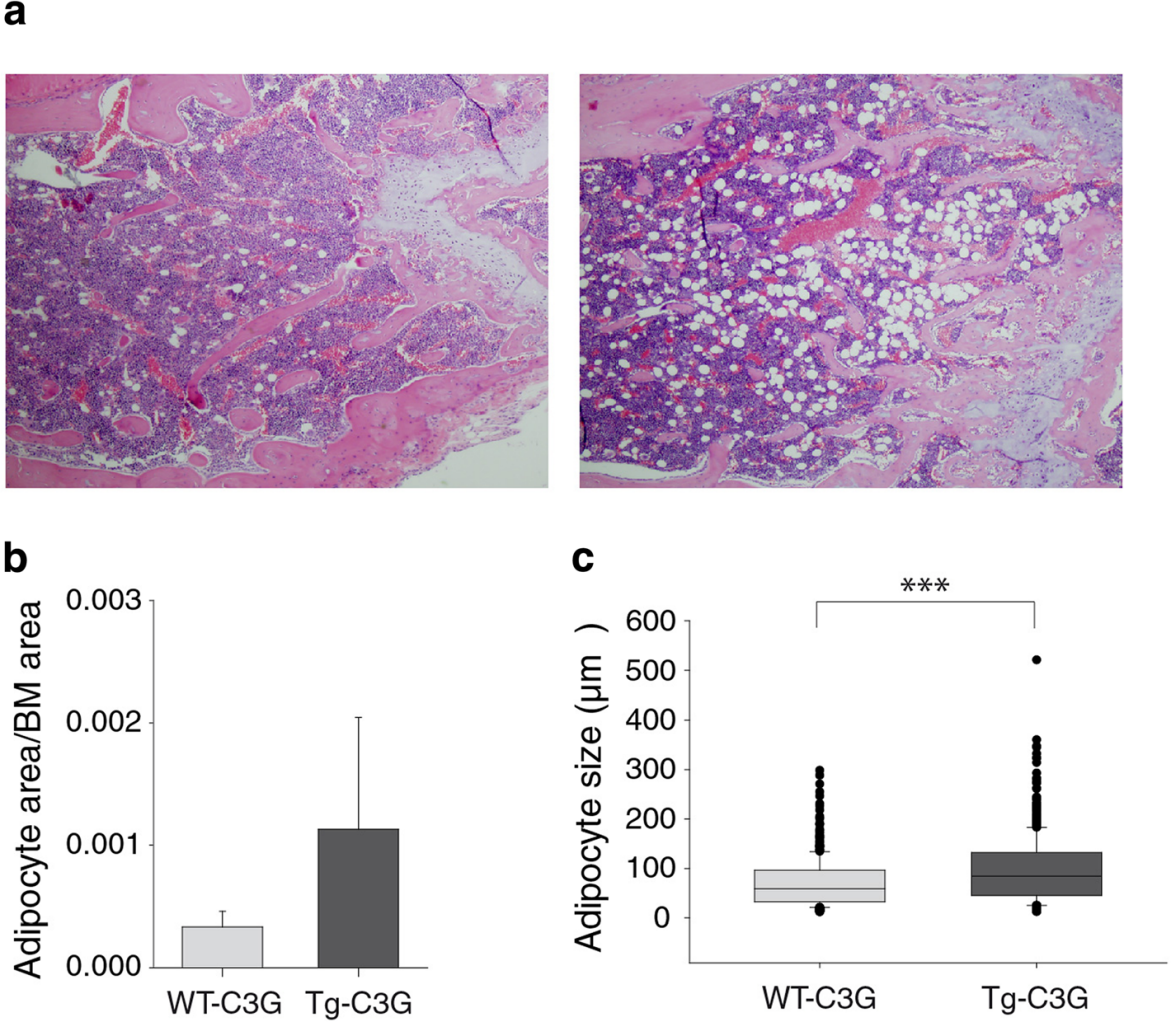
C3G induces adipocyte density in bone marrow after tumor implantation. B16-F10 metastatic melanoma cells (1 × 10^6^) were injected subcutaneously in Tg-C3G and WT-C3G mice. After 15 days of tumor growth, the bone marrow was harvested, fixed with 4% formaldehyde, embedded in paraffin and stained with H&E. **a** Representative images of bone marrow from femurs of transgenic and wild type mice. **b** Histogram represents the median ± SEM of the total adipocyte area relative to the bone marrow area. Mann-Whitney U test was done, but no significant differences were observed between Tg-C3G vs WT. **c** Box plot represent the median of the adipocyte size (μm2) of individual adipocytes. At least 4 different mice of each genotype were analyzed. Mann Whitney U test was done. ***p < 0.001

To further investigate the effect of C3G under pathological conditions, we monitored the number of platelets in Tg-C3G and WT-C3G mice 15 days after subcutaneous injection of B16-F10 melanoma cells. We did not observe differences, neither in the number of megakaryocytes in the bone marrow (Additional file 6: Figure S6), nor in the number of platelets in peripheral blood (data not shown). However, Tg-C3G showed a significant increase in the density and size of adipocytes in the bone marrow, as compared to WT-C3G mice (Fig. 8a-c).

## Discussion

In the present work, we have demonstrated that C3G plays a key role in the modulation of different stages of megakaryopoiesis leading to platelet formation. This includes colony formation of MK progenitors, acquisition of surface markers, changes in morphology, polyploidization, migration from the osteoblastic to the vascular niche and proplatelet formation. A role for C3G in other differentiation processes has been demonstrated, such as those leading to adipocytes [8], neuroblastoma cells [4], macrophages [7] and myotubes [9], which is probably a reflect of its essential function during embryogenesis [3].

K562 and HEL cells behave as pluripotent hematopoietic precursors, expressing features of both erythroid and megakaryocytic lineages when maintained under undifferentiated conditions. In fact, both cell lines can be induced to undergo erythroid or megakaryocytic differentiation depending on the stimuli. Thus, under PMA treatment, both cell lines exhibit characteristic features of megakaryocytes, including large size, presence of vacuoles and long extensions [16, 35, 36]. These same features were observed in non-stimulated K562 cells overexpressing C3G, suggesting that C3G could be involved in the modulation of morphological changes accompanying MK differentiation, presumably acting downstream of PKC. Moreover, the treatment with PMA for several days increased the expression of C3G in K562, and more notably in HEL cells, further suggesting the involvement of C3G in the actions of PMA that lead to MK differentiation. In this regard, we have previously demonstrated the participation of C3G in the PMA-PKC-Rap1 pathway leading to integrin αIIbβ3 activation in mouse platelets [21]. In agreement with a putative role for C3G in MK differentiation, expression of the megakaryocyte markers, CD41 and CD61, dramatically increased in K562 cells with stable overexpression of C3G, in the absence of any stimulus. Moreover, the levels of MK markers induced by C3G overexpression were similar, or even higher, than those induced by PMA in control cells. This indicates that C3G, per se, is capable of triggering the process by which these erythroid-like cell lines commit to the megakaryocytic lineage. In agreement with these findings, C3G expression is regulated by GATA-1, a transcription factor that is implicated in megakaryocyte differentiation.

However, C3G-silencing or ablation did not induce a decrease in the expression of these markers below the control levels, neither under basal conditions nor under PMA treatment. One plausible explanation is that the levels of these markers are very low in untreated cells, which makes very difficult to detect any further decrease. However, we cannot rule out the possibility of the existence of a compensatory mechanism, so that the absence of C3G would be countervailed by other Rap1-GEFs. A potential candidate could be CalDAG-GEFI, which is one of the most abundant GEFs in murine platelets and plays a significant role regulating their functions [37, 38]. However, so far there are no data about any potential role of CalDAG-GEFI in MK differentiation.

The acquisition of the megakaryocytic markers in these cell lines was linked to the downregulation of the erythroid ones, which is in concordance with the existence of a common precursor (MEP) for the erythroid and megakaryocytic lineages [39]. As a consequence, overexpression of C3G in K562 cells completely abrogated α-globin expression, including that induced by Imatinib (STI-571). Similarly, although to a lesser extent, the expression of GPA also decreased in the C3G-overexpressing K562 cells. In addition, an increase in the expression of GPA was evident in K562 cells upon C3G silencing or knockout, despite the lack of effect in the expression of the megakaryocytic markers. Similar results were observed in primary bone marrow cells from transgenic C3G and C3GΔCat mice, where a higher proportion of CD41/CD61 positive cells were found in Tg-C3G mice and a lower in Tg-C3GΔCat mice, compared to wild type animals. These findings, together with the greater number of CFU-MKs found in Tg-C3G BM cultures, support a role for C3G in the regulation of early stages of the erythroid/megakaryocytic commitment.

The analysis of the DNA content in C3G overexpressing and C3G silenced K562 cells indicate that C3G plays an important role promoting polyploidy in these cells. This correlates with the increase in nuclear size observed in C3G-overexpressing K562 cells. Thus, overexpression of C3G increases the proportion of polyploid cells, while its ablation induced a visible decrease. It should be highlighted that, although these results did not reach statistical significance, they were consistent in the two cell lines used and in both experimental conditions, i.e. C3G overexpression and C3G knockout. Moreover, a higher proportion of mature megakaryocytes (large CD41+ cells) with a DNA content ≥8n was also observed in the bone marrow of the Tg-C3G lineages as compared to WT, with no differences in Tg-C3GΔCat mice. These results corroborate the positive role played by C3G in the acquisition of polyploid features, which seems to be dependent on its GEF activity. The role of C3G in the regulation of ploidy was PMA-dependent, since no changes were observed under basal conditions (untreated clones). We have shown that treatment with PMA induced an increase in the phosphorylation of C3G in Y504. The phosphorylation of C3G in this residue plays an important role in its full activation, probably through the release of some auto-inhibitory mechanisms [33, 40]. It is plausible that an increase in C3G phosphorylation is required in later stages during MK differentiation, but not in the initial phases to induce the expression of the MK markers.

Additionally, overexpression of C3G in K562 cells produced an increase in the expression of the cell cycle inhibitor p21, which is known to be up-regulated during MK differentiation [32]. An upregulation of p21 induced by C3G overexpression also occurs in neuronal differentiation, which is mediated by the ERK1/2 pathway [4]. However, p21 levels in our C3G knockout K562 cells remained similar to those from control cells, even though these cells reach lower levels of ploidy. A plausible explanation is that the function of C3G in the induction of the endomitotic cycle requires other cell cycle regulators, in addition to p21. In fact, it has been shown that p21 knockout does not rescue the mitotic arrest during differentiation, indicating that its role in endomitosis is not essential [30]. Overall, these results suggest that C3G could be facilitating the exit of normal cell cycle and the entry into an endomitotic cycle.

The percentage of megakaryocytes in untreated mouse BM cells was similar in Tg-C3G, Tg-C3GΔCat mice and their controls (data not shown). These results indicate that, in contrast to the observed in the cell lines, C3G alone is not able to modulate the acquisition of MK markers in a more physiological context, requiring an additional stimulation, such as TPO, to potentiate this process. An important stage in MK differentiation is the interaction of megakaryocytes with the matrix, which regulates the migration of megakaryocyte precursors from the osteoblastic to the vascular niche. It has been shown that the interaction of type I collagen with immature megakaryocytes through its main receptors, integrin α2β1 and GPVI, inhibits the premature release of platelets [41, 42]. Our results showing a lower proportion of MKs bound to the osteoblastic matrix in Tg-C3G mice, compared to wild type mice, indicate that C3G overexpression decreases the interaction between the megakaryocytes and the endosteal niche. Interestingly, the proteomic analysis of platelets from Tg-C3G 2C1 mice and their controls, revealed a differential expression of several proteins involved in adhesion and migration, such as Integrin-linked protein kinase, Rho GDP-dissociation inhibitors, Cofilin-1, Tubulin β-1 chain or Actin-related protein 3 (unpublished observations from our group), which is in favor of a role for C3G in the regulation of adhesion and migration during MK maturation. This is reinforced by the observation that bone marrow explants from Tg-C3G mice contained megakaryocytes that migrate at a higher speed, covering a greater distance, being the catalytic activity of C3G responsible of this effect. Accordingly, we observed an increase in the number of proplatelet-forming megakaryocytes in our C3G transgenic mice. In contrast, C3GΔCat transgenic mice showed a reduced percentage of proplatelets, compared to their wild type. Therefore, we can conclude that C3G, through its catalytic, GEF domain, induces MK migration and proplatelet formation.

All this was not reflected in an increase in the production of platelets in peripheral blood. However, we observed a modest increase in the number of Tg-C3G platelets after the injection of TPO, in correlation with a decrease in Tg-C3GΔCat versus their respective controls. This result indicates that the Tg-C3G platelets appear to be more sensitive to TPO than the wild-type platelets, whereas the opposite applies to the Tg-C3GΔCat platelets. This correlates with the known role of C3G in the TPO-triggered signaling pathway [13]. In agreement with that, platelets from Tg-C3G mice showed higher platelet activation and aggregation in response to different platelet agonists [21]. In addition, Tg-C3G expression increases the capacity of platelets to induce angiogenesis and tumor metastasis [23].

Surprisingly, although tumor implantation did not alter the platelet count, a significant increase in adipose cells was found in the BM from femurs of Tg-C3G mice. This suggests that the forced expression of C3G in megakaryocytes under a stress condition (i.e. tumor implantation) could induce an adipogenic differentiation. Bone marrow adipocytes (BMAs) play important roles in hematopoiesis through the regulation of HSC function, and also participate in tumor development and metastasis [43, 44]. These functions are mediated by interactions with the BM microenvironment, including HSC-derived cells, such as macrophages and megakaryocytes [45]. Our hypothesis, supported by the role of C3G in the regulation of platelet secretion [23], is that, in the presence of tumor cells, C3G could enhance the ability of megakaryocytes to secrete factors that promote adipogenesis. Further studies will be conducted to address this hypothesis.

## Conclusion

In summary, our results unveiled a novel function of C3G as a promoter of megakaryocyte differentiation, maturation and proplatelet formation. This might be relevant, not only for physiological megakaryopoiesis, but also for pathological processes involving platelets, such as cancer or cardiovascular diseases. Our results are compatible with a scenario in which TPO triggers MK differentiation and platelet formation through the activation of a C3G-Rap1 signaling pathway. Future studies should aim at discerning the precise physiological and pathological function of this effect of C3G favoring megakaryopoiesis under specific conditions.

## Additional files

Additional file 1: **Figure S1.** C3G regulates the size and morphology of K562 cells. **a** Representative immunofluorescence confocal microscopy images of the indicated K562 clones stained with rabbit anti-C3G antiserum #1008 and DAPI. All clones express the GFP protein, encoded in the pSuper plasmid (pSuper.gfp/neo from Oligoengine). Images were obtained using a Leica TCS SP5 confocal microscope. Scale bars: 25 μm. **b** May-Grünwald-Giemsa staining of K562 clones with silenced C3G expression, untreated or treated with 20 nM PMA for 72 h. Scale bars: 10 μm. **c** Expression of CD41 and CD61 markers was analyzed by flow cytometry using specific fluorochrome-conjugated antibodies (CD41-APC and CD61-PE). Histograms represent the mean ± SEM of the fluorescence intensity (relative units) of CD41 and CD61 from at least 4 independent experiments of each clone. 2-way ANOVA and Holm-Sidak analysis were done. **p < 0.01. (JPG 1563 kb)

Additional file 2: **Figure S2.** Stable C3G silencing and knockout in K562 and HEL cell lines increase the expression of GPA. **a** The expression of CD41, CD61 and GPA markers was analyzed by flow cytometry using specific fluorochrome-conjugated antibodies (CD41-APC, CD61-PE and GPA-PE). Representative flow cytometry plots of untreated cells are shown. Histograms represent the mean ± SEM of the fluorescence intensity (relative units) of CD41, CD61 and GPA from at least 4 independent experiments of each clone, treated as indicated. 2-way ANOVA and Holm-Sidak analysis were done. *p < 0.05, **p < 0.01, ***p < 0.001. **b** Representative Western blots showing the decreased, or abrogated, expression of C3G in the three clones used. Values are relative to cells transfected with empty vectors. The expression of tubulin was used as loading control. C3G/Tubulin ratios are shown. **c** Analysis of CD61 and GPA mRNA expression by RT-PCR in the untreated K562 clones: pSuper-CT and pSuper-C3Gi. The numbers represent expression values relative to pLTR2-CT expressing cells and normalized against GAPDH, used as housekeeping gene. (JPG 578 kb)

Additional file 3: **Figure S3.** Negative controls of immunofluorescence Rap1 activation assay. The staining of Ral-GDS-RBD negative control was made without the Ral-GDS-RBD purified protein. Anti-GST and anti-p-C3G negative controls were made without the corresponding primary antibodies. Scale bars: 7.5 μm. (JPG 1284 kb)

Additional file 4: **Figure S4.** The RAPGEF1 locus shows binding sites for hematopoietic transcription factors. Alignment of the sequences conserved throughout the evolution in the C3G gene and in its flaking regions in opossum, rat, mouse, dog and cow, with respect to the human ortholog, obtained from the ECR browser database. The alignment is divided into 2 fragments representing the 3′ intergenic region and a large part of the gene (**a**) followed by the remaining part of the gene and the 5′ intergenic region (**b**). Peak intensity represents the degree of similarity, between 50 and 100%. In the upper part the human C3G gene is represented in blue. C3G transcript gives rise to two main splicing variants (a and b). Yellow arrows indicate the first exon of each variant. Exons are represented in blue, introns in salmon, intergenic regions in red, repetitive loci in green and untranslated regions (UTR) in yellow. The peaks corresponding to binding sites for specific hematopoietic transcription factors (HTF), are indicated by black arrows. **c** Representation of the HTF binding sites to the C3G gene conserved throughout evolution (up to mouse), and the regions in which HTF binding has been demonstrated by ChIP experiments (UCSC, Santa Cruz, California). HTFs whose binding has been demonstrated are represented as pins that indicate the DNA binding site. GATA-1 and GATA-2 sites are represented in purple, E2A sites in yellow, TAL-1 sites in blue and NFE2 sites in red. Black vertical lines show conserved transcription factor binding sequences. (JPG 3616 kb)

Additional file 5: **Figure S5.** Tg-C3G and WT-C3G platelets have a similar clearance. Histograms represent the mean ± SEM of the peripheral biotinylated platelets collected from Tg-C3G (**a**), Tg-C3GΔCat (**b**) and their wild type mice after 24, 48, 72, and 96 h of the NHS-Biotin injection. (JPG 170 kb)

Additional file 6: **Figure S6.** C3G did not modify megakaryocyte levels in bone marrow after tumor implantation. **a** Histogram represents the mean ± SEM of the percentage of CD41 and CD61 positive BM cells from femurs of the indicated genotypes, analyzed by flow cytometry with anti-CD41-APC and anti-CD61-PE antibodies. **b** Histogram represents the number of MKs per area of bone marrow. Data was analyzed by Ariol software. Mann-Whitney U test was done, but no significant differences were observed between Tg-C3G vs WT. (JPG 159 kb)

Additional file 7: **Video S1.** Representative video showing the mobility of megakaryocytes from an explant. The images of the videos were used to calculate the speed and distance covered by the megakaryocytes, which is shown in Fig. 6a. (AVI 6619 kb)

## Abbreviations

BM: Bone marrow
MEP: Megakaryocyte erythroid progenitor
MK: Megakaryocyte
PMA: Phorbol 12-myristate 13-acetate
Tg: Transgenic
TPO: Thrombopoietin
