# Supplementary figures and images for: C3G, through its GEF activity, induces megakaryocytic differentiation and proplatelet formation
Source: Cell Commun Signal. 2018 Dec 19;16:101. doi: 10.1186/s12964-018-0311-5 (PMC6299959; doi:10.1186/s12964-018-0311-5)

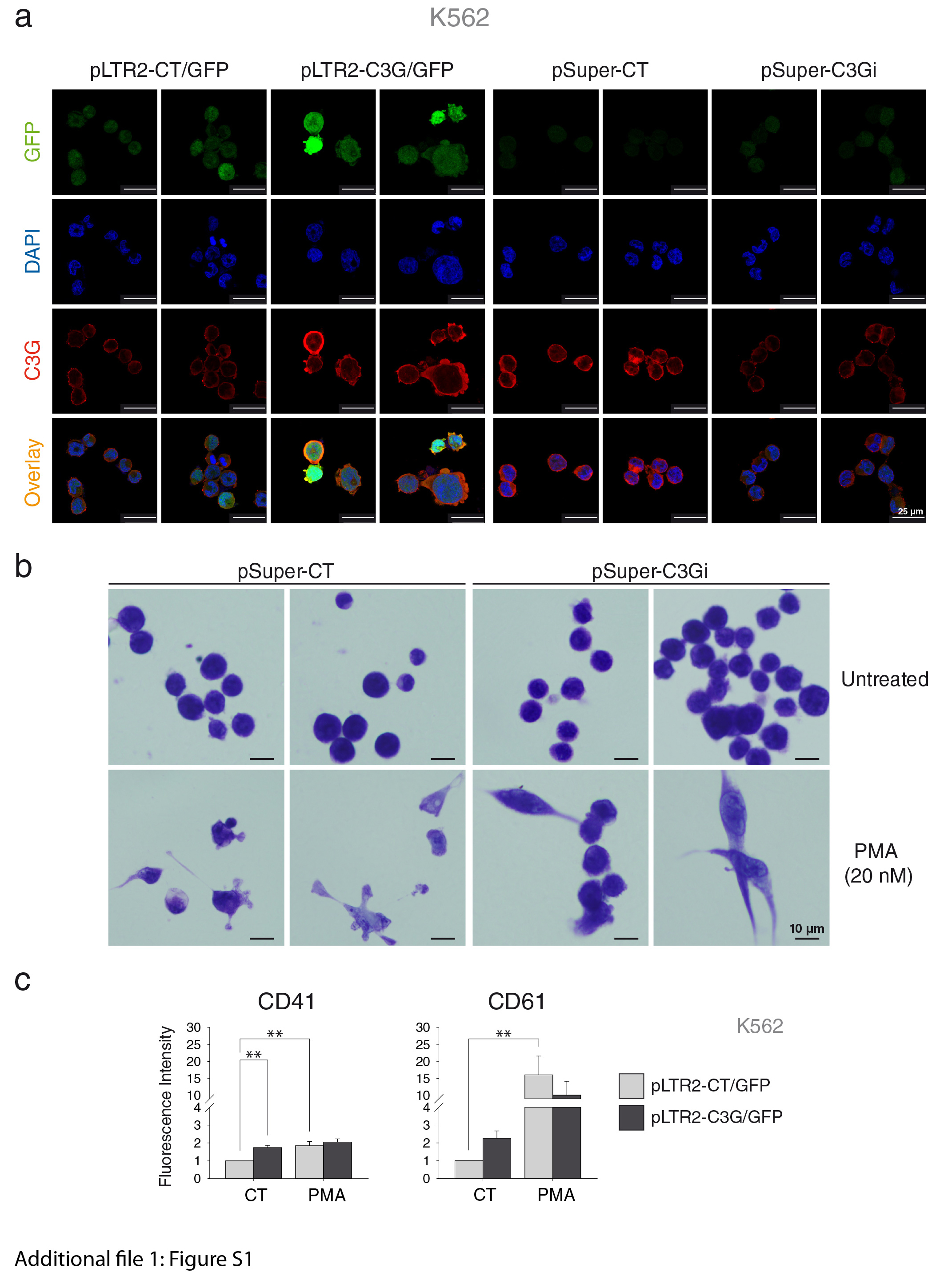

Supplement: Supplementary file 1 — Figure S1. C3G regulates the size and morphology of K562 cells. a Representative immunofluorescence confocal microscopy images of the indicated K562 clones stained with rabbit anti-C3G antiserum #1008 and DAPI. All clones express the GFP protein, encoded in the pSuper plasmid (pSuper.gfp/neo from Oligoengine). Images were obtained using a Leica TCS SP5 confocal microscope. Scale bars: 25 μm. b May-Grünwald-Giemsa staining of K562 clones with silenced C3G expression, untreated or treated with 20 nM PMA for 72 h. Scale bars: 10 μm. c Expression of CD41 and CD61 markers was analyzed by flow cytometry using specific fluorochrome-conjugated antibodies (CD41-APC and CD61-PE). Histograms represent the mean ± SEM of the fluorescence intensity (relative units) of CD41 and CD61 from at least 4 independent experiments of each clone. 2-way ANOVA and Holm-Sidak analysis were done. **p < 0.01. (JPG 1563 kb) [file 12964_2018_311_MOESM1_ESM.jpg]

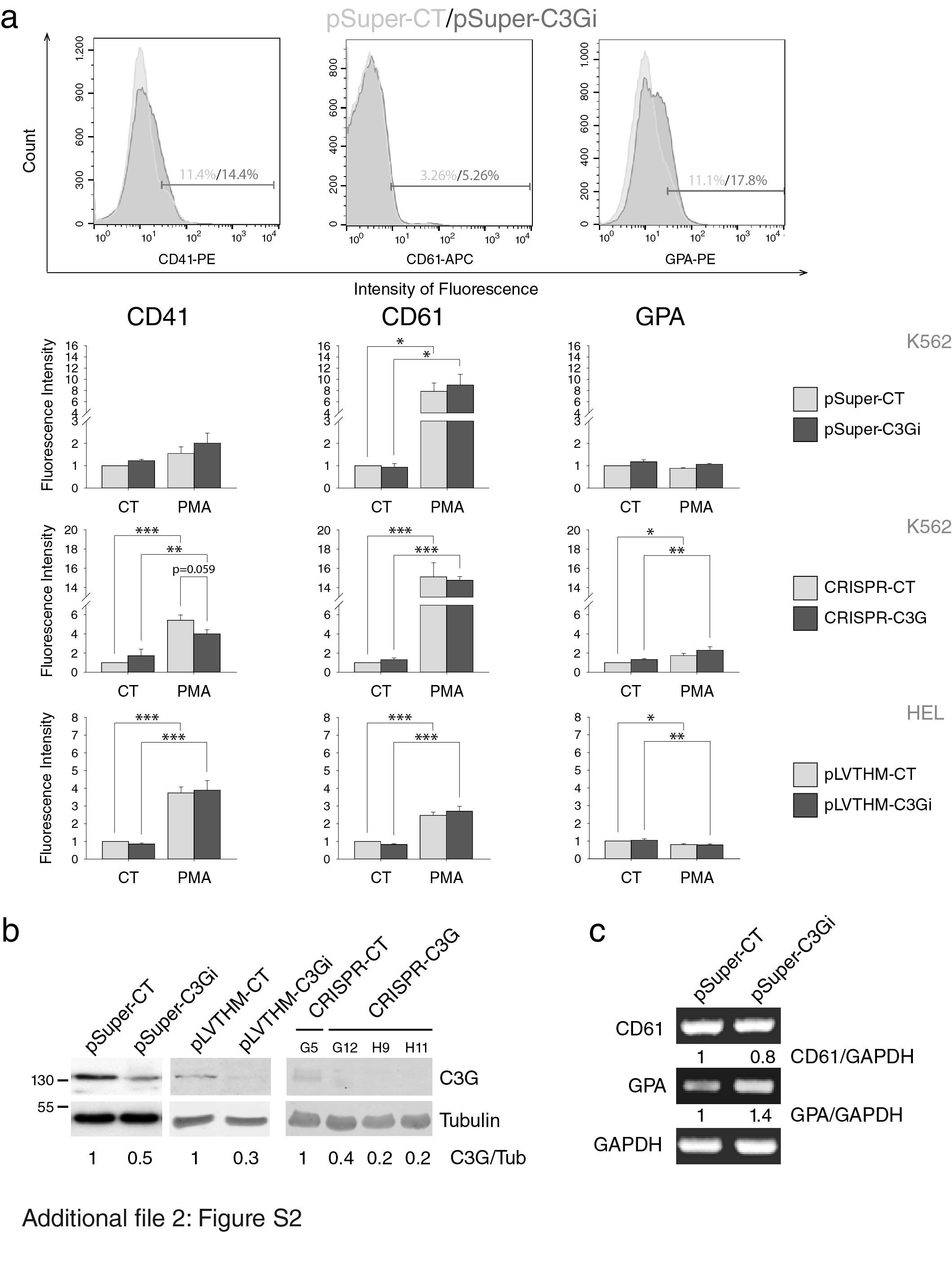

Supplement: Supplementary file 2 — Figure S2. Stable C3G silencing and knockout in K562 and HEL cell lines increase the expression of GPA. a The expression of CD41, CD61 and GPA markers was analyzed by flow cytometry using specific fluorochrome-conjugated antibodies (CD41-APC, CD61-PE and GPA-PE). Representative flow cytometry plots of untreated cells are shown. Histograms represent the mean ± SEM of the fluorescence intensity (relative units) of CD41, CD61 and GPA from at least 4 independent experiments of each clone, treated as indicated. 2-way ANOVA and Holm-Sidak analysis were done. *p < 0.05, **p < 0.01, ***p < 0.001. b Representative Western blots showing the decreased, or abrogated, expression of C3G in the three clones used. Values are relative to cells transfected with empty vectors. The expression of tubulin was used as loading control. C3G/Tubulin ratios are shown. c Analysis of CD61 and GPA mRNA expression by RT-PCR in the untreated K562 clones: pSuper-CT and pSuper-C3Gi. The numbers represent expression values relative to pLTR2-CT expressing cells and normalized against GAPDH, used as housekeeping gene. (JPG 578 kb) [file 12964_2018_311_MOESM2_ESM.jpg]

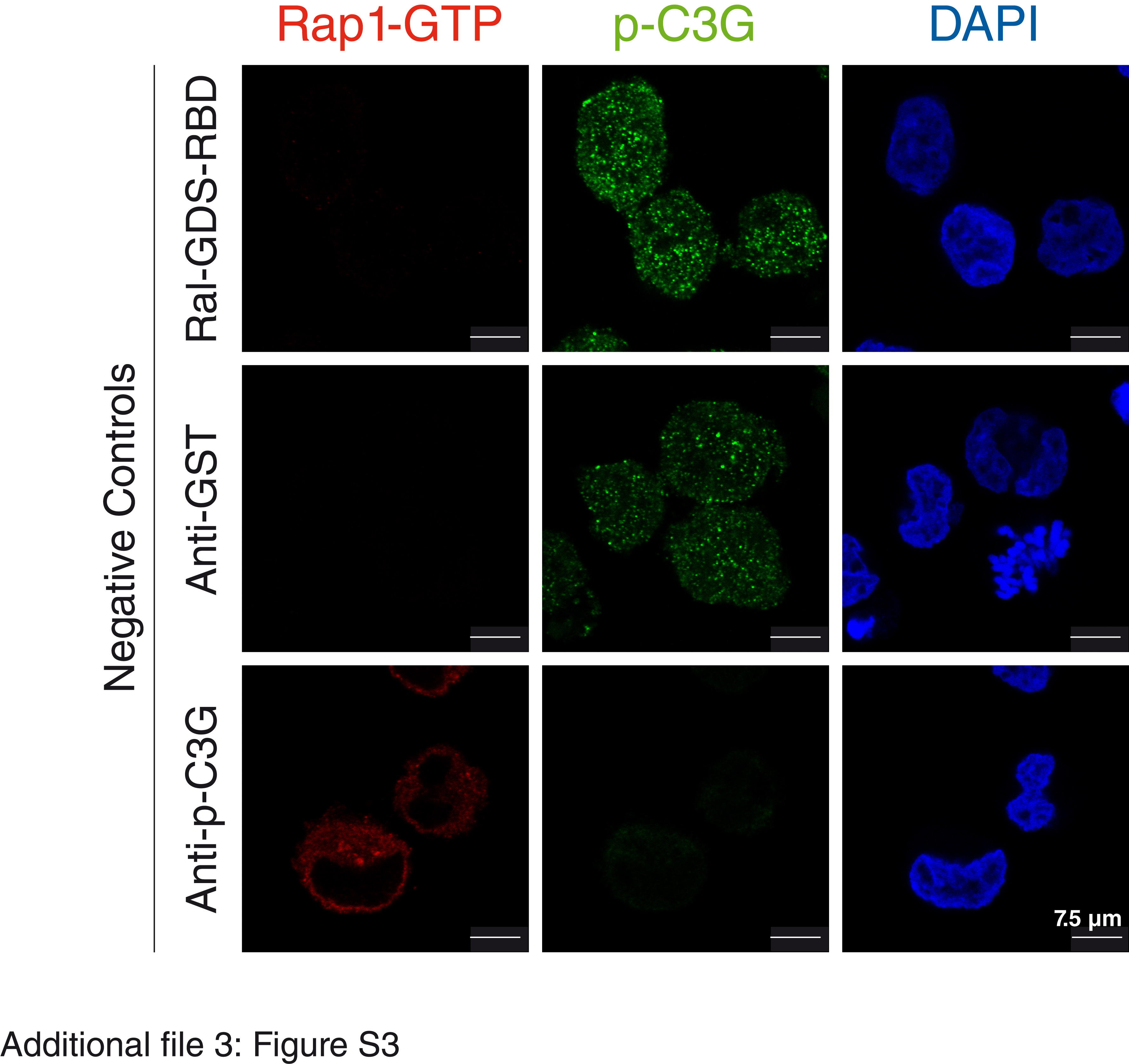

Supplement: Supplementary file 3 — Figure S3. Negative controls of immunofluorescence Rap1 activation assay. The staining of Ral-GDS-RBD negative control was made without the Ral-GDS-RBD purified protein. Anti-GST and anti-p-C3G negative controls were made without the corresponding primary antibodies. Scale bars: 7.5 μm. (JPG 1284 kb) [file 12964_2018_311_MOESM3_ESM.jpg]

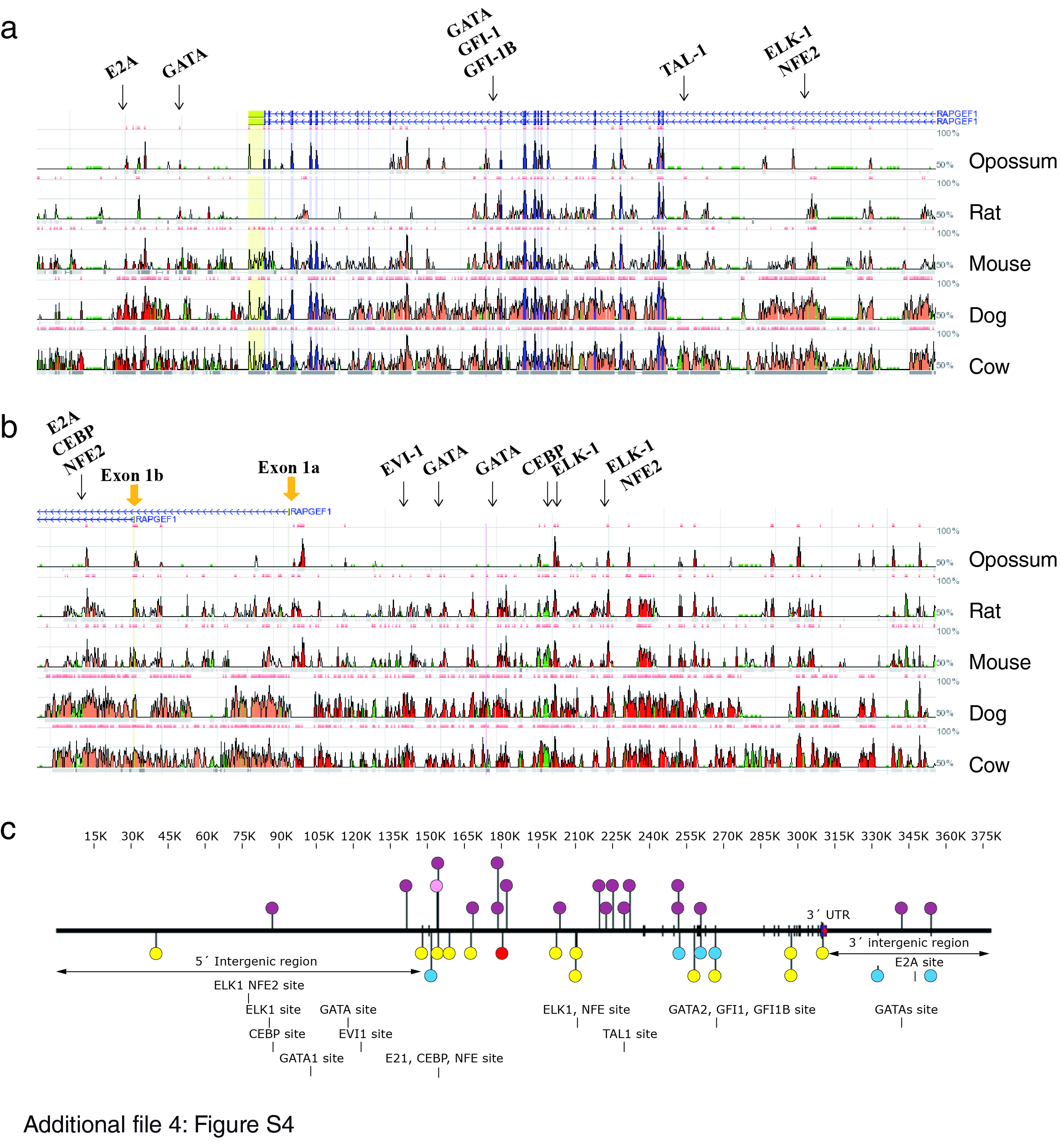

Supplement: Supplementary file 4 — Figure S4. The RAPGEF1 locus shows binding sites for hematopoietic transcription factors. Alignment of the sequences conserved throughout the evolution in the C3G gene and in its flaking regions in opossum, rat, mouse, dog and cow, with respect to the human ortholog, obtained from the ECR browser database. The alignment is divided into 2 fragments representing the 3′ intergenic region and a large part of the gene (a) followed by the remaining part of the gene and the 5′ intergenic region (b). Peak intensity represents the degree of similarity, between 50 and 100%. In the upper part the human C3G gene is represented in blue. C3G transcript gives rise to two main splicing variants (a and b). Yellow arrows indicate the first exon of each variant. Exons are represented in blue, introns in salmon, intergenic regions in red, repetitive loci in green and untranslated regions (UTR) in yellow. The peaks corresponding to binding sites for specific hematopoietic transcription factors (HTF), are indicated by black arrows. c Representation of the HTF binding sites to the C3G gene conserved throughout evolution (up to mouse), and the regions in which HTF binding has been demonstrated by ChIP experiments (UCSC, Santa Cruz, California). HTFs whose binding has been demonstrated are represented as pins that indicate the DNA binding site. GATA-1 and GATA-2 sites are represented in purple, E2A sites in yellow, TAL-1 sites in blue and NFE2 sites in red. Black vertical lines show conserved transcription factor binding sequences. (JPG 3616 kb) [file 12964_2018_311_MOESM4_ESM.jpg]

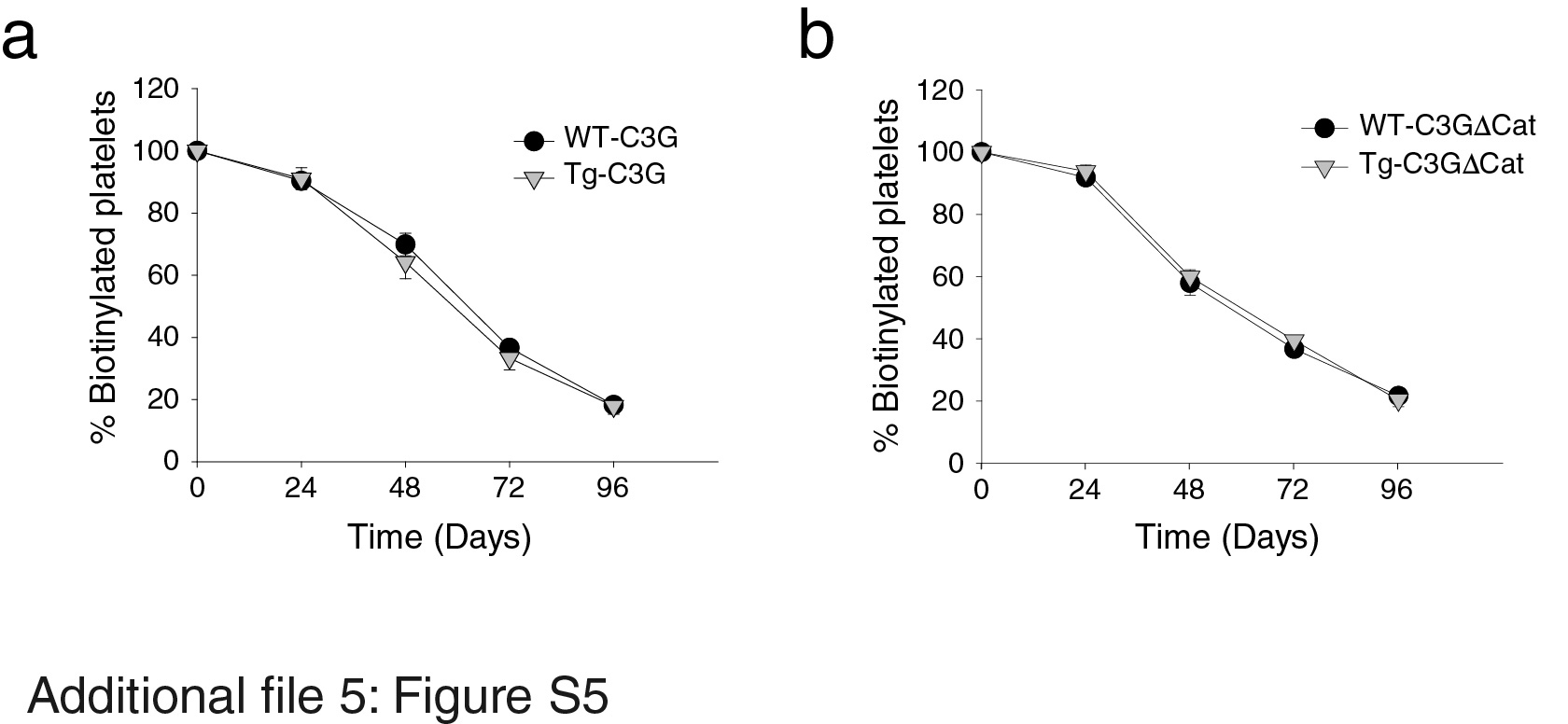

Supplement: Supplementary file 5 — Figure S5. Tg-C3G and WT-C3G platelets have a similar clearance. Histograms represent the mean ± SEM of the peripheral biotinylated platelets collected from Tg-C3G (a), Tg-C3GΔCat (b) and their wild type mice after 24, 48, 72, and 96 h of the NHS-Biotin injection. (JPG 170 kb) [file 12964_2018_311_MOESM5_ESM.jpg]

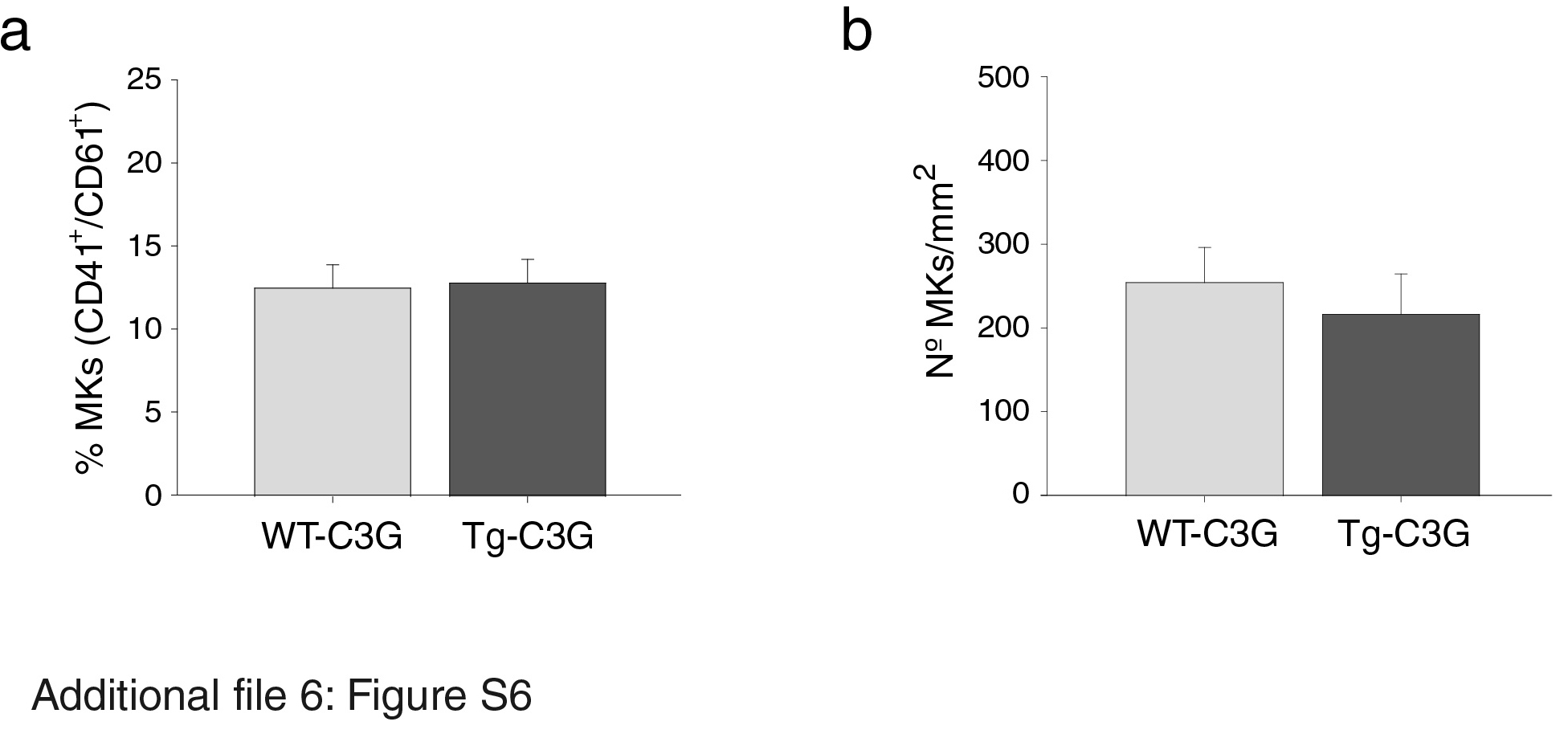

Supplement: Supplementary file 6 — Figure S6. C3G did not modify megakaryocyte levels in bone marrow after tumor implantation. a Histogram represents the mean ± SEM of the percentage of CD41 and CD61 positive BM cells from femurs of the indicated genotypes, analyzed by flow cytometry with anti-CD41-APC and anti-CD61-PE antibodies. b Histogram represents the number of MKs per area of bone marrow. Data was analyzed by Ariol software. Mann-Whitney U test was done, but no significant differences were observed between Tg-C3G vs WT. (JPG 159 kb) [file 12964_2018_311_MOESM6_ESM.jpg]
